# Domain adaptation in small-scale and heterogeneous biological datasets

**DOI:** 10.1126/sciadv.adp6040

**Published:** 2024-12-20

**Authors:** Seyedmehdi Orouji, Martin C. Liu, Tal Korem, Megan A. K. Peters

**Affiliations:** ^1^ Department of Cognitive Sciences, University of California Irvine, Irvine, CA, USA.; ^2^ Department of Biomedical Informatics, Columbia University Irving Medical Center, New York, NY, USA.; ^3^ Program for Mathematical Genomics, Department of Systems Biology, Columbia University Irving Medical Center, New York, NY, USA.; ^4^ Department of Obstetrics and Gynecology, Columbia University Irving Medical Center, New York, NY, USA.; ^5^ CIFAR Azrieli Global Scholars Program, CIFAR, Toronto, Canada.; ^6^ CIFAR Fellow, Program in Brain, Mind, & Consciousness, CIFAR, Toronto, Canada.

## Abstract

Machine-learning models are key to modern biology, yet models trained on one dataset are often not generalizable to other datasets from different cohorts or laboratories due to both technical and biological differences. Domain adaptation, a type of transfer learning, alleviates this problem by aligning different datasets so that models can be applied across them. However, most state-of-the-art domain adaptation methods were designed for large-scale data such as images, whereas biological datasets are smaller and have more features, and these are also complex and heterogeneous. This Review discusses domain adaptation methods in the context of such biological data to inform biologists and guide future domain adaptation research. We describe the benefits and challenges of domain adaptation in biological research and critically explore some of its objectives, strengths, and weaknesses. We argue for the incorporation of domain adaptation techniques to the computational biologist’s toolkit, with further development of customized approaches.

## Teaser

Research using biological data can benefit from domain adaptation modeling approaches, but it also carries distinct challenges.

## INTRODUCTION

In the computational biological sciences, we are interested in learning informative “truths” about biological systems through machine learning or similar quantitative modeling techniques (*1*). Contrary to “irrelevant” or “purely statistical” correlations, which find statistical idiosyncracies in data that do not reflect scientifically meaningful underlying patterns (e.g., when detecting COVID-19 from chest radiographs, a model may rely on confounding factors such as laterality markers or patient positioning, thus failing to generalize to new patients from other hospitals (*2*) and leading to misinterpretation of results within a single dataset), we expect such “truths” to generalize beyond a specific dataset or population, indicating that they offer a grounded biological meaning. However, collecting (and sometimes labeling) biological datasets is difficult, expensive, and time consuming, leading to many small but related datasets that are collected from different sources and under different environmental and experimental conditions (e.g., different laboratories, equipment, settings, humidity, etc.). For example, in the widely used Autism Brain Imaging Dataset (ABIDE), functional magnetic resonance imaging (fMRI) data were collected at multiple sites, which hindered the ability to directly aggregate data (*3*). Beyond creating challenges in data curation and metadata standards (*4*, *5*), this variability in the sources of small biological datasets creates different domains of data that have different statistical distributions.

While this variety is a strength that can facilitate discovery of generalizable truths, it also presents a major challenge to computational biology: Applying knowledge gained from one dataset (a source) to another (a target) will fail if the two datasets have highly divergent distributions—a phenomenon known as domain shift or data bias (*6*, *7*). In short, we cannot blindly apply a model (of any kind) trained on a source dataset collected under one set of conditions to new target data and expect it to perform effectively. In an age of open datasets and keen interest in adhering to FAIR principles (findability, accessibility, interoperability, and reuse of digital assets) to accelerate scientific discovery, it is increasingly urgent that we acknowledge the strengths and challenges of combining datasets.

**Fig. 1.**
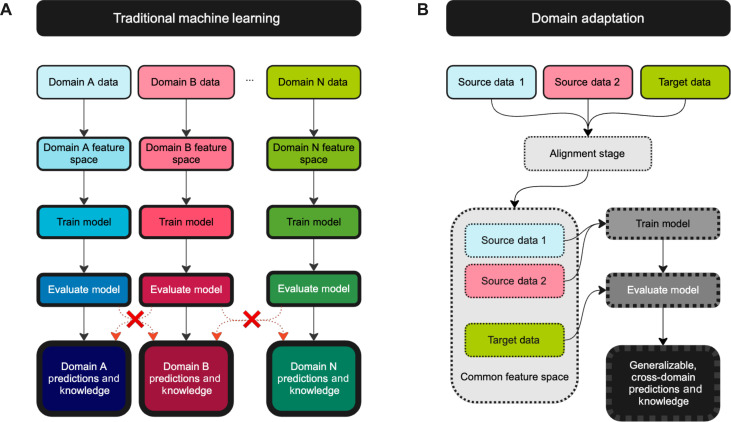
Diagrammatic overview of the machine learning pipeline and modifications needed to engage in transfer learning or domain adaptation (DA). (**A**) In traditional machine learning, each domain has its own model, trained on domain-specific features. This means that the model can make predictions about data from that domain, but transferring the model to apply it to other domains is typically difficult or even impossible (indicated by red Xs). (**B**) In transfer learning or DA, data from one or more source domains are aligned (denoted by dashed outlines) with those in the target domain to find common feature spaces with similar statistical distributions such that a single model can be trained on aggregate source domain data and evaluated on target domain. This process can produce generalizable knowledge that is not domain specific. Of note, in some cases, target data will only be used after the model has been trained and not in the alignment stage (*152*).

To best extract generalizable insights while making use of all collected data from varying sources—especially in biological disciplines where data are expensive—and to apply these insights to newly collected data, we must find how to best leverage the use of all existing and continuously growing small biological datasets (*8*). Here, computational biologists can borrow insights from machine learning to leverage transfer learning, which aims to use knowledge gained from learning a task on one dataset to perform a similar task on a different but related dataset, thereby transferring knowledge across datasets (*9*–*13*). More precisely, domain adaptation (DA), a subfield of transfer learning, has been developed to address this issue of different statistical distributions by aligning the distributions of the source and target domains (Fig. 1). Of note, while there are some similarities to “batch correction” often applied in high-throughput molecular measurements (*14*, *15*), the objective is different: DA aims to learn generalizable models across domains, while batch correction is primarily aimed at removing technical variation.

DA is more than just “lining up the features” and training a model on both datasets; not only is this often impossible to do (especially if features are unlabeled), but statistical differences between the domains can often guarantee that such a brute force aggregation is doomed to failure. Instead, through DA, a model is forced to learn domain invariant features, i.e., features that are common across all domains, such that the learned model can be generalized and thus perform relatively well on a separate target domain. Another benefit of DA is that the integration of multiple datasets effectively increases the sample size, allowing for improved inference of statistical signals. This allows better use of available data and resources, reducing the need to collect and annotate expensive data (*16*–*18*). Thus, in sum, it seems clear that applying DA to biological data can potentially mitigate small sample sizes within individually collected datasets, and through transferring knowledge to other domains can ideally find generalizable truths (Table 1).

**Table 1.** Specific benefits of using DA with biological datasets.

Problem	DA benefit	Example
Mitigate poor sample-to-feature ratios		
Complex biological systems often need to be modeled with many free parameters, while training samples remain quite few.	Integrate individual datasets to increase the number of training samples, providing a larger and more diverse dataset and preventing overfitting.	Combining fMRI datasets across individuals or scanning sites (*139*).
Transfer knowledge		
Some domains are poor in data due to either small sample size or missing labels.	Transfer knowledge from existing rich datasets to related, smaller datasets.	Transferring insights gained from MRI in adults to newborns (*155*); annotations from preclinical cell lines to more data-scarce clinical settings (*136*).
Find generalizable patterns		
In the age of big data sharing through FAIR principles, biological datasets are often composed of many different small cohorts collected from different laboratories and under different environmental and experimental conditions (*19*, *53*). These many smaller datasets drive models to finding patterns that turn out to be statistical anomalies or idiosyncracies unique to each dataset*.	Combining as much data as possible while minimizing statistical differences between domains can minimize the risk of models finding statistical idiosyncrasies rather than patterns shared across domains.	Finding cohort-independent generalities across multiple studies of the vaginal microbiome in preterm birth (*133*, *134*) or the gut microbiome in colorectal cancer (*156*, *157*) despite the variability in microbiome profiling (*154*).

*The statistical anomalies and idiosyncracies noted here are also related to two other related concepts: batch effects (*153*), when nonbiological artifacts change the distribution of the data for a subset of an experiment (e.g., plates for DNA extraction in microbiome cohorts (*154*); days or machines for MRI data collection), and batch confounding, when batches are associated with the outcome of interest.

However, DA is not a panacea, and computational biologists should be aware of the particular challenges of using such methods to analyze biological datasets. Compared to datasets typically used to train machine learning models (*19*–*22*), many “biological-scale” datasets are smaller in sample size, have many more features than samples, and have a complicated feature space (e.g., different numbers of features in each dataset, missing values, heterogeneous features, unique feature importance distributions, etc.). Therefore, while developing effective DA techniques that can work well with these small “biological-scale” datasets to find general truths about biological systems is highly desirable, it presents a specific set of challenges to machine learning research.

In this Review, we aim to critically discuss the benefits and challenges of applying current DA methodologies and frameworks to such biological datasets. To this end, we use the token examples of fMRI and microbiome datasets, two seemingly different disciplines in biology, to show the common considerations critical to developing effective DA techniques in such data. Our goal is to lay out the key components that require consideration in selecting an effective DA technique and highlight important areas of future methodological research in DA methods that can be maximally effective in biological datasets—especially as data sharing and metadata curation continues to mature. Our hope is that this discussion and synthesis will be of value both to biologists seeking to apply DA to their own data and to machine learning researchers driving state-of-the-art advances in DA methodology.

## CHALLENGES OF DOMAIN ADAPTATION IN BIO-SCALE DATA AND A PATH FORWARD

As briefly introduced above, successful application of DA to small datasets with complex features comes with substantial challenges—many of which stem from the very reasons we would want to use it in the first place. We next explore several of the most pressing limitations in greater detail, both to help researchers learn to evaluate DA approaches for appropriateness in their own research and to highlight deficiencies in current DA applications to biological questions, which may be alleviated through improved collaboration between DA researchers and computational biologists. We introduce and expand on several challenges below and summarize the challenges covered here in Table 2.

**Table 2.** Challenges of DA that are specific to biological datasets.

Challenge		Description
Poor sample-to-feature ratios		State-of-the-art DA approaches often require tens of thousands (or even millions) of samples to train [e.g. (*27*–*29*)], but biological datasets have a few dozens or hundreds of samples despite having thousands of features (*31*–*33*). DA models should be evaluated on biological-scale datasets [e.g., Office31 (*36*)].
Complex features	Missing values	Traditional DA models are not as often evaluated on data with many missing feature values, which is common in biological data [e.g., rare taxa in microbiome research (*56*–*59*)]. DA models should be evaluated in the context of missing data.
	Heterogeneous features	Biological data often have different features in different domains, only some of which are shared across domains (*61*). Traditional DA approaches often assume that features are shared and alignable (e.g., pixels with Cartesian coordinates), and in the face of poor labeling (*62*) or unavailable information about which features are shared (*12*) may simply deal with nonshared and non-alignable features by removing them from the DA model’s inputs. DA for biology should focus on targeting feature alignment in the context of some shared and some unique features across domains.
	Feature importance distributions	In traditional machine learning benchmark datasets, many features can be similarly important to the performance of a model (*40*–*43*), but in biological datasets, sometimes only a few features are very important. Thus, feature importance distributions are quite different in biological data than in DA benchmark datasets. DA models should be evaluated on datasets with varying feature importance distributions, ideally matching those found in biological data.
Data collection and preprocessing contributions		Biological data must be extensively preprocessed after primary data collection. Choices about which preprocessing steps to take, and which software packages to use, can meaningfully alter statistical distributions of feature behavior. Machine learning models thus can easily fall prey to preprocessing-induced statistical idiosyncracies (*75*), even when standardization efforts are made (*76*). DA approaches should be evaluated on their robustness to preprocessing choices for biological data.
Feature interpretability		Biological dataset features can be difficult to interpret: They are not simply pixel luminances at specific image coordinates, for example. Especially in the case of latent features found through DA (*83*–*86*) or simply dimensionality reduction techniques, care should be taken to integrate DA approaches with emphasis on interpretability to maximize their utility for biology.
Theoretical limitations		DA can only be successful if the source and target domains are *adaptable—*i.e., theoretically joinable (*89*–*92*). Adaptability (*89*, *91*) is highly understudied in biology, and failures of adaptability can lead to negative transfer, or cases where DA causes more harm than benefit (*89*, *90*, *93*). Methods development and empirical study are crucial to understanding theoretical limits on adaptability in biological domains.

### Number of samples and features

Most DA methods have been designed in the fields of computer vision, text mining, or language processing (*23*–*26*) with reference to—and evaluation on—large-scale text and image data, where there can be tens of thousands (or even millions) of samples available for training [e.g., MNIST, CIFAR10; (*27*–*29*)]. In contrast, the number of samples in biological datasets is often small, but they simultaneously have many features, a problem known as the curse of dimensionality (*30*). For instance, in a typical fMRI or microbiome dataset, we might only have a few dozens to hundreds of samples, while the number of features could exceed thousands (*31*–*33*). This imbalance between the number of samples and features can potentially lead to overfitting problems (*34*, *35*) or cases where the model performs well on training data but fails to make accurate predictions or conclusions from any other data; this, of course, hinders the effectiveness of DA techniques on biological datasets (*30*). There do exist several datasets typically used to benchmark DA approaches that may be somewhat closer in size to biological-scale data, including Office31 (*36*), which contains image data of objects collected from three source domains with different resolutions, for a total of 4110 images from 31 object categories (132 images per category). However, while one might hope that DA methods that have shown success on Office31 (*37*–*39*) could be useful for biological data with similar sample size per category, it must be acknowledged that many biological datasets have very different properties than imaging data (*40*–*43*), and are even smaller, with only several hundred training samples in total. There is a need for DA algorithm development to specifically target success in the face of fewer training samples.

### Differences in feature complexity

Simply checking that DA approaches can perform adequately on small datasets is unfortunately unlikely to be enough. Another barrier to applying DA approaches to biological data is that features in biological domains are inherently much more complex than those in image data. For example, in many machine learning datasets such as MNIST or Office-31, image data are essentially pixel luminance values in the RGB and alpha channels that can be relatively simple to aggregate with other source data, for example by resizing the image (*6*, *44*–*47*). In the case of biological datasets, however, the inherent complexity of features can substantially hinder our ability to aggregate different sources of data. For example, biological datasets often contain missing values (*48*–*51*) or have different numbers of features with unknown mapping between domains (*52*) (i.e., which features in a source are “the same” as which features in a target domain). They can also exhibit nonlinear relationships or interactions between features (*51*, *53*–*55*), and unique data preprocessing requirements for each source can substantially increase the complexity of developing DA techniques for biological datasets. In other words, in addition to feature-to-sample ratio and number of categories, we need to take into account the complexity and heterogeneity of biological domains before using DA techniques on biological datasets. This increased complexity stems from several sources, which we next discuss in more detail.

#### Missing values

Biological samples often contain many missing feature values. For example, microbiome data typically only consists of only a few taxa that are shared by most samples and even less so across cohorts. Many taxa are rare, a phenomenon known as zero inflation in statistics (*56*). In human neuroimaging, positron emission tomography or MRI scans combined with patients’ genetic information can help with early diagnosis of Alzheimer’s disease. However, the very common problem of missing values (i.e., not every subject has completed multimodality data) can impede the ability of these multimodal models to make reliable predictions (*57*–*59*). Missing data are less problematic in many traditional datasets used to train DA approaches, meaning that these approaches may not deal with missing data well; to be successful with biological data, DA algorithms need to adequately handle both small data and missing values.

#### Heterogeneity of features

Biological domains also often have different numbers of features, and the features also often do not lie in the same rank order across domains. For example, fMRI data from a given brain region will have different numbers of voxels from one human subject to the next, and the information represented, for example, in voxel 1 in person A is unlikely to functionally align with the information encoded by voxel 1 in person B. While functional alignment approaches have been developed (*52*, *60*), they do not explicitly perform DA operations. In microbiome research, it can be unclear whether particular taxa are the same across datasets, especially because, sometimes, the measurement techniques differ (e.g., taxa are characterized using different regions of a marker gene such that the same taxa might be represented by different features in different datasets). These examples are in stark contrast to most image-based DA approaches, which can exploit physical proximity of features (pixels) through spatial convolution or learn feature importance maps based on spatial features alone (e.g., the center of an image may often be more informative than the edges).

In addition, domains may have some overlapping features but also some nonshared (distinct) features—i.e., those that are specific to one domain but not the other (*61*). Current DA techniques may not be very effective on such datasets since domains may lack supplementary information such as labels (*62*) or information about matching features or samples between datasets (*12*). This limitation could force researchers to remove domain-specific features and hence lose the capacity of DA models to benefit from these unique features in the learning process. Ideally, DA for biology could benefit from a specific focus on both feature alignment (ideally unlabeled) and principled ways to deal with shared versus nonshared features.

#### Distribution of feature importance

In biological datasets, feature importance distributions can be more highly skewed than in many standard benchmarks used to test DA approaches. That is, in biology, a few features can be very important for the ultimate performance of a model; in contrast, in typical benchmark datasets, many features can have similar importance (*40*–*43*). This difference in skewness of feature importance distributions can lead to extreme challenges for many DA approaches such that DA models that succeed even on small “typical” benchmark datasets may fail in biological applications.

### Contributions of data collection and preprocessing procedures

Biological datasets often require extensive preprocessing after the data collection stage, which can be inconsistent across datasets or laboratories. Preprocessing can refer to either specific steps that may be used or not to clean, align, or otherwise modify raw data, or to the specific software packages used to accomplish logically similar goals [e.g., DADA2 or deblur for 16*S* ribosomal RNA (rRNA) amplicon data (*63*, *64*), fMRIPrep (*65*) versus AFNI (*66*, *67*) or FSL (*68*–*70*) for fMRI images (*71*)]. These choices can be made because of individual laboratories’ conventions or because of development of new software or algorithm versions that challenge reproducibility even within a given dataset. For example, in MRI data, it has been clearly demonstrated that software selection at multiple stages of processing, atlas selection (e.g., Desikan-Killiany-Tourville versus Destrieux versus Glasser), and idiosyncratic quality control procedural choices can strongly affect group- and individual-based inferences (*72*); in extreme cases, some later preprocessing stages can reintroduce nuisance covariates that were originally filtered out. Hence, choices regarding the stage of processing at which to perform DA could strongly affect the success of DA approaches (*73*). Such effects can also be exacerbated when full pipeline details are not included in publications to aid in reproducibility or when specific preprocessing steps are applied without appropriate attention to how they may alter statistical features specific to biological data [see (*74*) for a discussion relevant to microbiome data].

As a result, machine learning methods used in biology are typically limited to being highly context- and preprocessing-specific, requiring careful design and tailoring to test the desired hypothesis appropriately (*75*). This often occurs even despite targeted efforts in bridging this gap by the means of setting up standards in generating and preprocessing the data (*76*), since some laboratory- and individual-specific idiosyncrasies are wholly unavoidable. For example, in fMRI data correction for subject’s head movement, using different scanning sequences or scanners can introduce data shifts that make applying DA techniques even more difficult (*3*, *77*–*81*). Even preprocessing methods meant specifically to correct for batch effects in microbiome research can introduce pipeline-specific factors (*82*), which may be easily overlooked. Such preprocessing idiosyncracies can thus exacerbate or interact with other batch effects, including introducing or altering interdependencies among features (*53*).

### Interpretability of features and feature spaces

Interpretability is an important aspect of biological research, in contrast to at least some other machine learning applications. However, alignment steps in DA, which often require finding a latent representation of data by projecting the domains into a shared feature space (*83*–*85*), are frequently carried out by machine learning and deep learning methods. This means that DA in biological data inherits the same problem that plagues machine learning more broadly: failures in interpretability due to the black-box nature of these machine learning and deep learning methods. The shared feature space is particularly challenging to interpret (*86*) because it is defined as a latent space that bridges two or more domains rather than the latent space defined by one domain alone. Therefore, DA research can and should aim particularly at understanding how input features are related to the common feature space when using these methods (*87*, *88*).

### Theoretical limitations of DA

It is also important that we discuss a critical theoretical limitation of DA, especially as it might affect biological data. The primary driver of DA’s potential success is the adaptability between the source and target domains (*89*, *90*)—essentially, the theoretically maximal ability of an ideal model to jointly model them (*91*, *92*). Failure of adaptability is thus a potentially fatal concern. While considering that additional source domains provide the benefits of a larger and more diverse sample set (or additional labels), these domains might have inherently different distributions of features or different joint distribution with the labels, which could mean that applying DA might ultimately bring more cost than benefit (*90*). In these worst-case scenarios, applying DA can result in what is known as negative transfer, which is when the application of knowledge from a source domain negatively affects the performance of a model in a target domain (*89*, *93*). For instance, Wang and colleagues (*93*) applied a domain-adversarial neural network (*94*) to transfer knowledge from product images as source domain to real-world images as target domain but found that the models’ accuracy on the target domain decreased by 10% because of divergence in lighting, angles, and photo backgrounds between domains. Crucially, the potential for negative transfer can be amplified when working with biological data due to its already-heterogeneous nature and the smaller sample size of each dataset, and due to unknown adaptability between biological domains. Therefore, it is imperative that the adaptability of the particular biological datasets in question be explicitly quantified or estimated before applying DA methods. Unfortunately, while there exist a few methods to quantify adaptability between domains (*89*, *91*), analysis in the context of different biological subfields is exceedingly rare. The development of adaptability analysis methods thus may be a fruitful and critical area of future research into DA application to biological datasets.

## CONSIDERATIONS FOR SELECTING AND APPLYING DA APPROACHES

Despite the challenges noted above, even in their current state, DA approaches can still provide benefit in biological data at this critical expansion of data sharing and open science practices in biology. However, there are a great many methods to choose from. How should a scientist select the best DA approaches for their own datasets or scientific questions? In this section, we outline specific considerations for biologists in selecting and applying DA approaches in their own research.

We begin this section by presenting a formal definition of domain and DA. We then present a taxonomy that can be useful in gaining a better understanding of what to search for in the literature. In this Review, we focus on the primary subcategory of DA that addresses data bias or covariate shift; this DA subcategory tries to align shifts in the feature spaces between domains (or the change in the marginal distribution of data samples across domains). Other specialized subcategories of domain shift include label shift (*95*), which indicates that different domains contain different number of labels for each class, and concept shift (*96*), in which the data distribution remains the same but the conditional distribution changes [i.e., $P_{s}\left(y|X\right)\neq P_{t}\left(y|X\right)$]. Interested readers should refer to these surveys (*97*, *98*) for a comprehensive overview of the different types of shifts in the DA field.

### What is a domain?

A domain can be defined as D={χ,P(X)}, where χ is a feature space, $X=\left\{x_{1},x_{2},\ldots ,x_{n}\right\}$ is an instance set with $x_{i}$ denoting a given feature, n denotes the number of features or dimensions in the data (e.g., in fMRI data voxel activities or taxa in microbiome data), and P(X) denotes the marginal probability distribution of all samples in that dataset. This formal definition is typically used in discussions of DA across a wide variety of disciplines (*99*, *100*).

### The terminology of DA

**Fig. 2.**
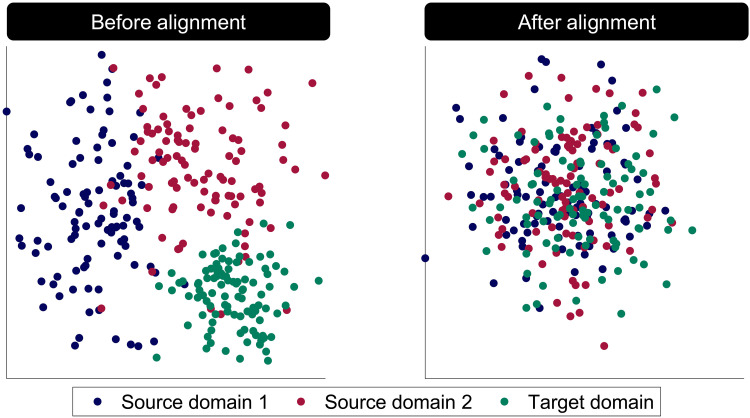
A cartoon representation of source and target domains before and after alignment. In this cartoon, features vary in their values along two dimensions, and each domain’s features take on a different mean and covariance. Unless the domains are aligned, these differences could both obscure other meaningful variation in the data that are shared across domains and prevent models trained on one domain from generalizing to another.

For a specific domain, we define the task (e.g., predicting what image a subject is looking at from neuroimaging data or predicting a disease state from microbiome composition) as T={y,f(·)}, where y denotes the labels to be predicted and f(·) denotes a decision function [i.e., the posterior probability distribution of P(y∣X) of the joint distribution P(X,y)] that needs to be learned to map input features to the corresponding labels. Given these definitions, DA is faced with the following problem, in which the distributions or relative alignment of features across domains are different but the task remains approximately the same. Thus, a DA problem with covariate shift can be formally defined as follows

$$P\left(X_{s_{1}}\right)\neq P\left(X_{s_{2}}\right)\neq \ldots \neq P\left(X_{s_{k}}\right)\neq P\left(X_{t}\right)$$

$$T_{s_{1}}\approx T_{s_{2}}\approx \ldots \approx T_{s_{k}}\approx T_{t}$$

where s denotes the source domain, t denotes the target domain, k is the number of source domains, P(X) is the marginal distribution of a specific instance set in a given domain, and T is the task performed in each domain. Here, the goal of DA is to improve the performance of target decision function $f{\left(\cdot \right)}_{t}$ in target domain $D_{t}$ by leveraging the information from source domain $D_{s}$ and decision function $f{\left(\cdot \right)}_{s}$ (which is learned on the source domain after the source and target domains are aligned). In other words, DA intends to adapt the model(s) trained from a source (or sources) to a different, but related, target dataset. It does this by aligning the distributions of features and samples belonging to different domains so that the models emphasize learning domain invariant features that are not dependent on a specific dataset (Fig. 2). The methods by which DA accomplishes this alignment differ depending on algorithm specifics; interested readers can refer to the Supplementary Materials for details, in which we catalog a number of different algorithms and their various applications.

### A taxonomy of DA

In general, when undertaking a DA analysis, we should consider three main factors:

1) The data used to train a model may be collected from multiple sources or just from a single source.

2) Depending on the availability of labels in the target domain, we might choose supervised, semi-supervised, or unsupervised models.

3) The feature spaces in the source(s) and target domains can be homogenous, meaning that they have the same dimensionality and “meaning,” e.g., feature A in source 1 represents the same “type” of information as feature A in source 2, or heterogenous, meaning that the feature spaces may differ in terms of dimensionality and/or meaning.

In the following, we discuss these three factors in more detail. Table 3 also shows a summary of these categories accompanied by mathematical annotations.

**Table 3.** Difference among traditional machine learning, transfer learning, and various kinds of DA. ML, machine learning; DA, domain adaptation. χ represents feature space, and P(X) is the marginal distribution of instance set X, T denotes the performed task, and f(·) is the decision function to map each sample to the corresponding label. s denotes the source domain, t denotes the target domain, and k is the number of source domains.

	Categories | definitions	Domains, $D=\left\{\chi ,P\left(X\right)\right\}$ and tasks, $T=\left\{Y,f\left(\cdot \right)\right\}$	Verbal description
Traditional ML versus transfer learning	Traditional ML	$D_{s}=D_{t}$ and $T_{s}=T_{t}$	When the source (i.e., training set) and target (i.e., test set) have the same distribution and the task is exactly the same.
	Transfer learning (TL)	$D_{s}\neq D_{t}$ or $T_{s}\neq T_{t}$ or both	When the source and target domains have different distributions or the performed task on source and target are different, or both.
Single- versus multisource DA	Single-source DA	$P\left(X_{s}\right)\neq P\left(X_{t}\right)$ and $T_{s}\approx T_{t}$	When there is only one source domain and the marginal distribution of the feature space between source and target domain is different. The task in the target domain is similar to that in the source domain.
	Multisource DA	$P\left(X_{s_{1}}\right)\neq P\left(X_{s_{2}}\right)\neq \ldots \neq P\left(X_{s_{k}}\right)\neq P\left(X_{t}\right),$ and $T_{s_{1}}\approx T_{s_{2}}\approx \ldots \approx T_{s_{k}}\approx T_{t}$	When there are multiple sources available that can have different distributions, and when these distributions differ from that of the target domain. The task is similar across all domains.
Supervised, semi-, or unsupervised	Supervised	$P\left(X_{s}\right)\neq P\left(X_{t}\right)$, with all target labels	When source and target domains are both labeled.
	Semi-supervised	$P\left(X_{s}\right)\neq P\left(X_{t}\right)$, with some target labels	When source is labeled but target is partially labeled.
	Unsupervised	$P\left(X_{s}\right)\neq P\left(X_{t}\right)$, with no target labels	When source is labeled but target is not labeled.
Homogeneous versus heterogeneous	Homogeneous DA	$P\left(X_{s}\right)\neq P\left(X_{t}\right)$ and $\chi_{s}=\chi_{t}$ and $T_{s}\approx T_{t}$	When the feature spaces have the same dimensionality and the same meaning.
	Heterogeneous DA	$P\left(X_{s}\right)\neq P\left(X_{t}\right)$ and $\chi_{s}\neq \chi_{t}$ and $T_{s}\approx T_{t}$	When the feature spaces have different dimensionality or different meanings.

#### Single versus multisource

In selecting a DA method, one question you will want to ask is how many domains are present. As mentioned above, DA techniques can be divided into two categories of “single source” and “multisource” (*101*). In single-source DA, the source domain is usually labeled, while the target domain belongs to another domain that has a different distribution (*13*, *85*). Single-source DA is simpler than multisource DA since there are only two distributions of data—source and target. Therefore, single-source DA is a good technique when there is enough data available in both the source and target domains to effectively train a model that can perform well on the target domain (*38*, *102*–*104*).

However, in modern real-world data sharing initiatives, most biological data come from many sources (*105*, *106*), and using these data to their full extent can facilitate novel insights. Therefore it is advantageous to develop models that leverage all available sources. This problem can be addressed through multisource DA, which aims to combine multiple sources of labeled data to make predictions about a similar task on a target dataset (*101*, *105*, *107*, *108*). A naive way to solve this problem is to combine multiple sources into one big source domain and then approach the problem as a single-source DA (*101*, *109*). However, these methods can show very limited improvement in performance—and sometimes even worse performance—in comparison to using only one source (*110*), specifically stemming from challenges of aligning the sources to begin with. Another way to tackle this problem could be to train a model on each source independently, apply each trained model to the target domain, and then vote for the “correct” label in the target domain based on the prediction across sources (*111*). One could also attempt to first find domain-invariant features among all source and target domains (*112*) or use a two-stage alignment technique that first tries to find domain-invariant feature spaces for each source-target pairing and then align model outputs across these spaces (*110*). In all cases, though, multisource DA is more challenging than single-source DA—a problem made worse by the particular characteristics of biological data, as discussed above.

#### Supervised versus semi-supervised versus unsupervised

It is also important to assess what kinds of labels are available for your data, across all the domains you need to align; this will dictate whether you should select a supervised, semi-supervised, or unsupervised DA method. These labels have been applied in varying ways (*13*, *101*, *113*–*115*). Here, we have chosen a categorization based strictly on the usage of target labels: In unsupervised DA, no label is available in the target domain (*38*, *85*, *116*, *117*); in semi-supervised DA (*118*–*120*), some labels are available to use; and in supervised DA, labels in the target domain are available for most samples (*97*). Although most DA techniques in existing literature focus on unsupervised DA (since it is often used for the purpose of annotating unlabeled data in the target domain), in the case of biological data, any of the supervised, semi-supervised, or unsupervised scenarios is possible. This is because the primary goal of DA in biological settings is to uncover insights about biological systems that generalize across domains. Thus, even when labeled data are available in the target domain, one can still benefit from using DA techniques on different datasets to find generalizable patterns across domains.

#### Homogeneous versus heterogeneous

Last, it is important to understand how the features are related across your different domains. DA can be divided into two categories based on the relationships between these features: homogeneous or heterogeneous (*97*, *99*, *101*). In homogeneous DA, the source and target domains have the same feature space, $\chi_{s}$*=* $\chi_{t}$, but the data distributions of instances of these feature spaces are different, $P\left(X_{s}\right)\neq P\left(X_{t}\right)$. That is, feature 1 in domain 1 represents the same meaning as feature 1 in domain 2—for example, they both represent a specific voxel at a specific coordinate in the brain or represent the same microbe. (Note that $\chi_{s}$*=* $\chi_{t}$ means that the feature space in both domains is homogenous, but if $X_{s}$*=* $X_{t}$, then, it means that $X_{s}$ and $X_{t}$ are identical datasets such that there is no difference between the source and target datasets at all.) In heterogeneous DA, conversely, the feature space is related but different between the domains. Many DA techniques that have been developed so far tend to focus on homogeneous DA (*84*, *121*–*129*). For instance, the source data could be the fMRI data obtained from a subject with one scanner and the target domain is the fMRI data obtained from the same subject with the same protocol but a different scanner. Alternatively, different domains could contain gut metagenomic sequencing data from different studies aligned against the same reference database. Addressing the domain shift in a homogeneous DA problem is relatively simpler since it is possible to perform the feature alignment directly on the original instances of the domains without the need to project them into a common feature space.

Unfortunately, however, most biological datasets are heterogeneous in nature (*51*, *53*) since these data are collected in different laboratories, under different environmental and experimental conditions, and sometimes even for answering different but related questions. In other words, neither the feature spaces nor the marginal distributions are the same [i.e., $\chi_{s}\neq \chi_{t}$,] $P\left(X_{s}\right)\neq P\left(X_{t}\right)$]. As a result, biological datasets very often have different feature dimensionalities, and, sometimes, these features even have different labels or come from different modalities of data collection (e.g., fMRI versus another neuroimaging modality like electroencephalography). For instance, the fMRI data from the brains of two individuals have different numbers of voxels (features), which also are not meaningfully aligned across individuals with respect to their functional properties (e.g., voxel 1 in person A is unlikely to encode the same information as voxel 1 in person B)—even when the scanner, protocol, and performed task are exactly the same.

### Case studies and practical examples

Given the nature of most biological datasets, which often contain limited samples and originate from many different sources, the most common DA setting in this field is multisource heterogenous DA settings. For instance, aggregating fMRI data from multiple subjects or even multiple sites (*130*–*132*) can be considered a multisource heterogeneous DA. It is multisource because the data are coming from multiple subjects or multiple sites with different MRI scanners, and it is heterogeneous because the number of voxels (i.e., features) from each subject and the information they represent is different. (Note that the number of voxels can be equated through spatial normalization to a standardized template, but this does not address that each voxel will still represent different information across individuals.). In the microbiome field, integration of data from multiple microbiome datasets in order to predict a phenotype on a held-out study (*133*–*135*) is once again multisource and heterogeneous, as data are often amplicons of different regions of the 16*S* rRNA gene. To illustrate the utility of existing DA approaches and explore their categorization with the taxonomy discussed above, here, we select several methods to discuss in slightly more detail (summarized in Table 4).

**Table 4.** Case studies and their categorization according to our DA taxonomy.

Method	Goal	Single or multi source?	Supervised, semi-, or unsupervised?	Homogeneous or heterogeneous?
PRECISE (*136*)	Predict patients’ drug Response based on preclinical datasets	Multi	Supervised	Homogenous
Adversarial inductive transfer learning (AITL) (*137*)	Predict drug responses on small and hard-to-obtain gene expression data	Multi	Supervised	Homogenous
WENDA (*138*)	Predict a human’s age using DNA methylation data, which are known to differ across tissues	Multi	Unsupervised	Homogenous
Li and colleagues’ (*3*)	Improve classification accuracy of autism diagnosis by detecting biomarkers in resting-state fMRI Autism Brain Imaging Data Exchange (ABIDE) datasets (*139*) from multiple sites	Multi	Supervised	Homogenous
Deep cross-subject adaptation decoding (DCAD) (*142*)	Learn common spatiotemporal patterns within a source fMRI domain (person) to generate labels for another person	Single	Unsupervised	Heterogeneous

One DA method, the PRECISE method (*136*), has been used to predict patients’ drug response based on available preclinical datasets such as cell lines and patient-driven xenografts (PDXs). To achieve this, the authors first extracted factors from cell lines, PDXs, and human tumors using principal component analysis (PCA). Then, they aligned these subspaces from human tumor data with preclinical data using geometric transformations and extracted common features associated with biological processes followed by training a regression model using consensus genes and validated with known biomarker-drug associations to accurately predict drug response in patients. In this study, DA was homogenous, as the features (genes) in the source and target domains were the same; multisource, as various source domains were used (i.e., cell lines), and supervised, as the labels of all samples were used.

Another method, Adversarial Inductive Transfer Learning (AITL) (*137*), similarly aims to use largely available source domains such as cell lines and clinical trials to predict drug responses on small and hard-to-obtain gene expression data from patients. To this end, researchers first used a feature extractor network to map the source and target into a common feature space. This mapping aimed to alleviate the domain shift by using a global discriminator to learn domain-invariant features. Then, these domain-invariant features were used to build a regression model for the source task (i.e., predicting median inhibitory concentration) and a classification network to make predictions on the target task (i.e., predicting whether there is reduction in the size of the tumor). This study aimed to address both prior and covariate shifts in the source and target domains. The data used in this study came from multiple heterogeneous sources including thousands of cell lines from different cancer types. Last, the target samples were labeled. This study can thus be characterized as a multisource and supervised heterogeneous (i.e., drug response is categorized differently between preclinical and clinical settings) DA scenario.

Other methods such as WENDA (*138*) (Weighted Elastic Net for unsupervised DA) aim to predict a human’s age using DNA methylation data, which are known to be different across different tissues. WENDA aims to use the available DNA methylation data from some tissues (source domains) to predict the age of the human subject using DNA methylation from a different tissue (target domain) by giving more importance to features that are more robust and behave in a similar fashion across source and target domains. In this study, data from 19 different tissues with chronological age ranging from 0 to 103 years old were used as the source domain. The target domain came from 13 different tissues, with chronological age ranging from 0 to 70 years old. In the application of WENDA, the source domain remained unchanged, while each tissue type was viewed as a distinct target domain. This thus represents a multisource, unsupervised, homogenous DA scenario.

In another study, Li and colleagues (*3*) propose a multisource DA approach by using resting-state fMRI ABIDE datasets (*139*) from multiple academic sites (UMI, NYU, USM, and UCLA). Their goal was to improve the classification accuracy of autism diagnosis by detecting biomarkers. In this study, the feature space, denoted as χ, was extracted features from fMRI sites such that $\chi_{i}=\chi_{j}$, with i and j representing different institutions (the data can be spatially normalized across participants by warping to MNI space). From this perspective, this problem is a homogeneous DA scenario. Subsequently, the authors used a Mixture of Experts (*140*, *141*), combining multiple neural networks—each of which is specialized in solving a specific task—to improve the overall performance of the model, and adversarial domain alignment methods to minimize the discrepancies between the domains, and successfully demonstrated the advantage of using federated DA techniques in using multisite fMRI dataset to classify autism. In addition, they were able to reveal possible biomarkers in the brain for autism classification. Therefore, in this framing, this can be considered as a multisource and supervised homogeneous DA problem.

Last, Gao and colleagues (*142*) proposed the deep cross-subject adaptation decoding (DCAD) method: a single-source, unsupervised, and heterogeneous DA technique. DCAD uses a three-dimensional (3D) feature extraction framework using 3D convolution and pooling operations based on volume fMRI data to learn common spatiotemporal patterns within a source domain to generate labels (*142*). Subsequently, an unsupervised DA method minimizes the discrepancy between source and target distributions. This process considers different subjects as different sources and aids in the precise decoding of cognitive states (in working memory tasks) across subjects. To validate the approach, they applied task-fMRI data from the Human Connectome Project (*143*) dataset. The experimental outcomes revealed exceptional decoding performance, achieving state-of-the-art accuracy rates of 81.9 and 84.9% under two conditions (four brain states and nine brain states, respectively) during working memory tasks. In addition, this study demonstrated that unsupervised DA effectively mitigates data distribution shifts, offering an excellent solution to enhance cross-subject decoding performance without relying on annotations.

## FUTURE DIRECTIONS

### What is missing from DA approaches in biological applications?

Despite these exciting successes, continued development of DA approaches tailored to the challenges of biological data is critically needed. This is especially important in light of the increasing availability of curated open datasets, complemented by increasing metadata standardization (*4*, *5*). We thus hope that the machine learning community will continue to develop techniques that can address relevant limitations of biological datasets, including:

1) Models must be able to capture the nonlinear and complex patterns in biological systems, ideally with minimal or no assumptions. Therefore, many linear-based or parametric DA techniques (usually focused on some sort of predetermined transformation from source to target domain) might not be adequate. See the Supplementary Materials for detailed descriptions of some existing DA techniques, many of which rely on such predetermined parametric assumptions. We recommend a concerted research program to catalog the successes and failures of existing DA approaches with respect to different types of biological datasets, with attention to the impact of predetermined parametric assumptions.

2) Ideally, we want to use DA to find the underlying mechanisms of biological phenomena rather than simply aggregating data for automatic annotation. Unfortunately, many existing techniques are primarily developed for addressing automatic annotation of unlabeled data. Therefore, to fully unleash the power of DA in biological systems, we must focus on methods that seek to find domain-invariant features that are common across datasets. This usually happens by mapping all domains into a common feature space. We recommend that DA research for biological application should prioritize discovery of latent or shared spaces between domains and ideally those which are “interpretable” or “explainable.” [“Interpretability” or “explainability” in machine learning may be defined as “how well a human could understand the decisions in the given context” (*144*).]

3) This domain-invariant mapping should be done using methods that work with limited data in individual cohorts. Although deep learning models are great tools to uncover highly nonlinear and complex relations in data with no specific assumptions, they often require many samples. Recently, simpler neural network architectures such as TRACE (*145*) and Fader networks (*78*) have shown promise with small fMRI datasets. However, many of the powerful neural network architectures such as generative adversarial networks might not be suitable for biological datasets as they usually require vast amounts of data (*146*, *147*). We recommend that DA research focuses on performance under data-scarce regimes, including explicit truncation of training datasets to evaluate DA methods under highly undesirable sample-to-feature ratios.

4) While some methods do exist to quantify adaptability between domains (*89*, *91*), limited attention has been paid to how such methods may fare in biological contexts. We recommend that DA research develops adaptability assessment methodologies with specific focus on biological datasets.

In sum, it is incumbent upon us in the biological disciplines to challenge machine learning research to design more flexible and broadly applicable DA methods that can perform under the constraints of real-world biological datasets. An important step toward this goal will be to test and evaluate existing approaches on our own data and on data available through broad and consistently annotated shared data repositories, to comprehensively explore and categorize their current shortcomings. Thus, we hope that, with the help of the topics discussed in this Review, researchers in biological disciplines will feel empowered to try out existing DA approaches and to help catalog their successes and shortcomings, which can then support the efforts of DA researchers to maximize the utility of such methods for biology.

If you would like to use DA techniques to augment your own data processing pipeline, we urge you to begin by gaining a comprehensive perspective on your data using the definitions and taxonomy described above. For example, How many sources do you have available? What is the sample size in each source? Do these sources contain equal amounts of features? If not, what are the nature of features in each source? Are these features in each source known and have a label? What task are you trying to achieve? Depending on the answers, you can choose the appropriate DA approaches and set about examining their successes or failures. We hope that the tools and information provided in this Review will encourage you to do so and to report your findings so that iterative improvements in DA approaches can be made to best serve our fields.

### Promises for the future

In this piece, we have focused on human neuroimaging (specifically fMRI) and microbiome sciences as token examples to speculate the potential promises of DA in computational biology as a whole. We hope that these selected case studies have helped to show off the potential of DA in numerous and varied biological disciplines, from electrophysiology, multi-omics, DNA sequencing, and single-cell RNA sequencing to protein localization—all of which face similar challenges in data collection and labeling to the case study fields discussed here. Differences in equipment, experimental setup, or even individuals can lead to a shift in the distribution of data, even when the task is identical. In all cases, however, our goal as researchers and clinicians is to go beyond domain-specific or dataset-specific models to find domain-general and informative “truths” about biological systems.

Thus, DA could be extremely useful to aggregate diverse biological datasets available across the Open Science Framework, OpenNeuro, Neurosynth, Dryad, CEDAR, and more in search of meaningful and even clinically relevant outcomes (*148*–*151*). However, much work is needed to address the existing challenges. It is the intention of this paper to help and facilitate these processes by bringing more awareness of DA and the need to develop new techniques that are compatible with the limitations of biological datasets in order to make it accessible to biologists. If we are successful in identifying the challenges of performing DA on biological data, we are optimistic that DA and transfer learning methodologies can greatly benefit biologists.

## Supplementary Materials

### This PDF file includes:

Supplementary Text Table S1 References
