# Supplementary material for: Domain adaptation in small-scale and heterogeneous biological datasets
Source: Sci Adv. 2024 Dec 20;10(51):eadp6040. doi: 10.1126/sciadv.adp6040 (PMC11661433; doi:10.1126/sciadv.adp6040)
Supplement: Supplementary file 1 — Supplementary Text Table S1 References [file sciadv.adp6040_sm.pdf]

Science Advances
AAAS

Supplementary Materials for

Domain adaptation in small-scale and heterogeneous biological datasets

Seyedmehdi Orouji *et al.*

Corresponding author: Email: Tal Korem, tal.korem@columbia.edu; Megan A. K. Peters, megan.peters@uci.edu

Sci. Adv. **10**, eadp6040 (2024)
DOI: 10.1126/sciadv.adp6040

This PDF file includes:

Supplementary Text
Table S1
References

Overview of supplementary material

Here we provide more detail on specific domain adaptation methods than offered in the main text. The purpose of this section is to provide a greater degree of detail for the interested reader, as well as to suggest further reading through the works cited.

Domain adaptation through adding or selecting features

Some DA methods discover the best way to transfer between domains by adding, deleting, or otherwise weighting features from each domain differently. In the following subsections, we describe some representative methods in detail.

Adding features: feature augmentation / feature replication

The *feature augmentation* or *feature replication* strategy aggregates and transforms the source and target domain features together into an augmented feature space for use during model training. For example, Frustratingly Easy Adaptation (FEDA)(*124*) maps the augmented source and target feature space by duplicating the features into three vectors, $\chi_s = (X_s, X_s, 0)$ and $\chi_t = (X_t, 0, X_t)$, to regulate the trade-off between source/target and general weights for the classifier to learn. Daumé and colleagues(*158*) provide a semi-supervised extension of the FEDA method by forcing the source and target domains to agree on the unlabelled data to leverage unlabeled data in the target domain for model training.

Although relatively easy to implement, one should be cautious when applying FEDA methods to small-scale biological data. First, biological data have high dimensionality to begin with, so duplicating features will make the dimensionality of the data even more troublesome -- especially when multiple domains are intended to be leveraged simultaneously. Domain-independent feature reduction methods such as PCA can be applied, as was done by Schneider and colleagues(*159*), to dramatically reduce the number of features. However, this approach's performance did not substantially differ from the results of simply concatenating the source and target domains. Furthermore, FEDA methods only work with homogenous datasets, while biological data is often heterogeneous across domains. Therefore, a proposed extension of FEDA that deals with data heterogeneity by using two projection matrices P and Q, $\chi_s = (PX_s, X_s, 0_{dt})$ and $\chi_t = (QX_t, 0_{ds}, X_t)$, has been found to be generally more useful when dealing with biological data(*9*).

Selecting or weighting features

Data selection domain adaptation strategies include methods in which the respective local geometrical structures of data in both source and target domains remain unchanged. In other words, these methods select only the informative and relevant features or samples to use in training and testing, without implementing any transformational changes to the data itself. Data selection often occurs in conjunction with and before other transformation and alignment techniques (see next section), though using it on its own can offer a beneficial and simpler approach to bridging source and target domains in some situations.

Structural correspondence learning (SCL)(*160*) is an example of this data selection strategy. SCL first defines a set of frequently occurring and diverse *pivot* features (i.e. features that behave the same way for discriminative learning in all domains) on the unlabeled data from both domains, and then estimates the pivot features' covariances with non-pivot features to learn a mapping function between source and target. Another version, a manifold-based technique(*161*), employs a method termed Statistically Invariant Sample Selection (SISS) to select landmark samples from both domains based on the pairwise Hellinger distances between the samples' distributions. In some cases, SISS has been shown to be more effective than assigning non-binary weighting to samples(*162*). Of course, the drawbacks of discarding samples that do not meet these criteria when working with small-scale biological data -- when samples are already too few -- are obvious given the discussion in the main text. However, the tradeoffs between (a) using only a few informative samples, thus risking not having enough samples, and (b) selecting all samples thus risking *negative transfer* (i.e. applying knowledge from a source domain will negatively affect the performance of the model in a target domain), should be judged on an empirical and case by case basis.

Domain adaptation through parametric transformations

Another important domain adaptation strategy concentrates on *alignment*. There are different ways to align domains, including label information or dependency structure and correspondence of features. Overall, the merit of alignment techniques is that most of them do not require that label information is available for the target domain, and some also reduce the dimensionality in a way that takes into account both the source and target. Many of these methods thus also discover a (often lower dimensional) shared (sub)space between the source and target domain, rather than a transformation that maps one domain directly onto the other. A major critique of these methods is thus that they often result in less interpretable features -- and feature interpretability is a critical objective in many biological studies. Here, we briefly discuss several parametric alignment-based approaches to DA.

(Note that domain alignment can be done in a parametric or non-parametric way, depending on how the loss function is minimized; in this section we focus specifically on parametric alignment, with nonparametric approaches to both feature selection and alignment discussed in **Domain adaptation through nonparametric feature selection and transformation approaches (neural networks)**, below.)

Correlation based alignment

Canonical correlation analysis (CCA)(*163*) is a classical technique used to maximize the correlation between two sets of vectors -- or in the case of DA, the correlation between the feature distributions across two domains. CCA is particularly useful in heterogeneous DA problems where the goal is to find a feature transformation to bridge the heterogeneous feature spaces via finding a subspace that is shared between two domains(*164*). However, CCA by definition finds *linear* combinations of two domains, and so cannot work where a nonlinear feature subspace is desirable(*165*–*167*). Fortunately, advanced implementations of CCA, such as Kernel CCA

(KCCA)(*165–167*), can be employed to find non-linear combinations of shared feature representations across domains.

Another correlation-based approach, Correlation Alignment (CORAL), is a simple and fast, yet powerful, technique to minimize domain shift by aligning the second-order statistics (covariance) of two distributions(*136*). CORAL tries to minimize distances between domains using the original feature spaces rather than lower-dimensional subspaces. The first step is to remove feature correlations in the source domain (i.e. 'whitening'), and then 'recolor' the source's features by the target domain's feature covariances. The advantage of the CORAL method is that it is incredibly simple and fast -- for example, it can be implemented with four lines of code in MATLAB -- and yet is still very effective in aligning the domains. However, we note that this method is limited to aligning homogeneous domains.

Geometric transformation based alignment

Several methods assume that transformations must take on a specific functional form, potentially based on field-specific knowledge. For example, suppose one wishes to functionally align fMRI data across multiple human subjects to study shared cognitive characteristics and improve the samples-to-features ratio. Some methods to accomplish this goal use rigid-body geometric transformations because they assume that specific regions of the brain (e.g. ventral temporal cortex) encode similar features across domains, but that these features are not labeled (i.e., the coordinate system of voxels that represent specific features are not aligned). These unlabeled features across subjects must be aligned into a common feature space across multiple subjects without warping the feature space – i.e., using only translation, scaling, and rotation. Here, subjects are considered as different source domains, where the dimensionality and order of voxels (features) are different across subjects. These characteristics thus suggest a heterogeneous multi-source DA problem.

One such method, hyperalignment(*63*, *168*), assumes that the shared feature space is high-dimensional. This method also assumes that data from all source domains share the same feature space that is also anchored by the same temporal variance (e.g. all domains/participants have watched the same sequence of a movie). Therefore, under the assumption that the features in each domain follow a shared trajectory in some shared feature space while anchored in time, it is possible to rotate the individual feature spaces into a common feature space. (Other variants of hyperalignment technique have been proposed(*70*, *169*).) This rotation is done through Procrustean transformation – an orthogonal transformation that minimizes Euclidean distance between features across domains. This model finds hyperalignment parameters that will map each source into a common feature space of voxel responses. This method implements two main assumptions that must be considered before applying it to other datasets. First, there exists a feature space that is common between all domains, and second, there exists a linear transformation that can map the voxel pattern of multiple domains into a common feature space such that it can minimize the distance between two features in two domains. Therefore, hyperalignment might also be useful for aligning heterogeneous multi-source DA problems where these two assumptions are satisfied, especially when there is also the opportunity to exploit temporal anchoring.

Other parametric transformations

Probability distributions lie on a Riemannian manifold, and there are some alignment-based methods that exploit this fact. One method(*170*) utilizes manifold alignment without the need for any pairwise correspondence information between the source and target. More concretely, for a given source $X_s = \{x_i, \cdots, x_m\}$ of feature dimension p and target $X_t = \{x_j, \cdots, x_n\}$ of feature dimension q, the method computes functions α and β to map source and target domains to a new lower dimensional space so that $\alpha^T x_i$ and $\beta^T x_j$ can be directly compared in order to minimize the loss function. Another similar method(*72*) expands manifold alignment to work on multiple domains with heterogeneous datasets and leverages the labels to align the domains rather than the often-inaccessible correspondence information (i.e. instances in one dataset that correspond to, or are in some way equivalent to, instances in another dataset).

Other methods can be seen as less rigid versions of the methods discussed above. For example, like hyperalignment, the Shared Response Model (SRM) was originally developed to aggregate fMRI data across many subjects to evaluate cognitive states across groups rather than within individuals(*171*). However, unlike hyperalignment, SRM projects subjects' data into a shared *lower-dimensional* feature space, and thus relaxes the assumption of rigid-body transformation and precisely shared (but unlabeled) features within a given brain region that are shared across participants. The shared space, S, can be calculated as follows

$$min\ W_{i,S} \sum_{i=1}^{m} \left|\left|X_i - W_i S\right|\right|^2$$

where $||.||$ is Frobenius norm, and X_i, W_i are the fMRI responses and bases for the participant i, respectively. This is under the orthogonality assumption of W_i such that $W_i^T W_i = I_K$ where K is the dimension of shared feature space that is chosen by the experimenter.

Likewise, in the microbiome field, specific frameworks have been developed to model the processes that generate variability between different studies or batches - for example by modeling experimental variability as multiplicative bias that affects the measured taxonomic abundances(*172*). These functional forms can then form the basis for field-specific domain adaptation techniques. One such method is DEBIAS-M(*143*), a microbiome-specific domain adaptation method that realigns sources by inferring their underlying processing biases.

Domain adaptation through nonparametric feature selection and transformation approaches (neural networks)

Sometimes, it is not possible to define *a priori* the type of transformation that might be appropriate to align domains or select features. In this case, it is advantageous to turn to neural networks,

which can discover and approximate any parametric function. Neural network based DA techniques thus typically have a feature extractor section in their architectures, as well as relying on standard objective functions to align domains. This section will focus on introducing various neural network architectures and the type of biological problems that they may be well–suited for.

Adversarial based

Adversarial based neural network methods usually incorporate a discriminator component in their architectures. Unlike discrepancy based methods that try to align features by minimizing a specific statistical distance measure between domains (e.g. the CORAL, hyperalignment, or SRM methods above), adversarial methods try to "fool" a discriminator until it cannot distinguish which data is coming from which distribution(*122*, *139*, *173*, *174*), and as a consequence, the network will learn domain-invariant features(*129*, *175*).

Adversarial-based methods can be separated into two categories: adversarial generative and adversarial discriminative. Adversarial generative methods, usually based on Generative Adversarial Networks (GANs), were originally designed to solve problems where there is interest in generative models(*176*). For instance, Xu and colleagues(*121*) used GAN loss in the objective function in order to minimize the discrepancy between each source domain and target by using multi-adversarial learning. Although GANs can create fascinating visualizations, they are not optimized for discriminatory tasks and are limited to domains where the shift between distributions is small(*174*). Moreover, these networks usually need many training samples, which of course is an issue in biological datasets, and hence might not be a good choice for biological data. On the other hand, adversarial discriminative methods aim to mitigate the negative effects of domain shift by learning a discriminative representation of the source and target domains(*122*, *174*) without the need for a generative component. These methods, however, have also typically been used on image data where there are tens of thousands of examples available, and so require further examination to explore whether they can be suitable for small biological datasets.

To utilize these methods, however, one has to use a feature extractor section in the architecture of the model in order to map the original input spaces into a (usually) lower-dimensional space. A simple yet effective architecture of adversarial-based DA is the domain-adversarial neural network (DANN)(*49*), developed by Ganin and colleagues, which uses a gradient reversal in order to maximize the loss of a categorical classifier and hence to ensure that features in the two domains are similar.

Autoencoder based

Autoencoder (AE) networks are unsupervised learning algorithms that consist of an encoding and a decoding section, which enable them to learn hidden representations of an input(*177*). The encoding section of an autoencoder usually uses a nonlinear function in order to discover features in a bottleneck (the dimensionality of the bottleneck is usually lower than the initial input) that are informative enough to be used by the decoding section of the network to reconstruct the original input. In DA, it is possible to learn domain invariant features by sharing the same encoding section across multiple domains. The unsupervised nature of these AE networks makes them a good

candidate to discover domain-invariant features even in the case of unlabeled or sparsely labeled source domains.

Previously, AEs have been successfully used to extract domain-invariant features. For instance, stacked denoising autoencoders (SDAEs) have been used before to extract high-level features that are common across source and target domains(*178*). Therefore, a classifier trained on these high-level common features using the labels available in the source domain can also perform well on the target domain. Similarly, Bousmalis and colleagues(*179*) developed the Domain Separation Network (DSN) using two encoding sections. First, a shared encoding network between source and target domains learns the shared representation across domains, and second a private encoder learns domain-specific features. Then the decoding section will use both domain-invariant and domain-specific representations to reconstruct the input samples. Finally, the authors trained a classifier only on the shared representation so the classifier can also perform well on the target domain. As another example, Ponimova and colleagues(*88*) successfully developed the Fader network, which used a convolutional autoencoder to address the heterogeneous nature of multi-site fMRI data to increase the sample size and extract domain-invariant features across multiple subjects. Although the total number of samples were 1000, they were collected from 19 sites which means the average available data from each site was 52 samples. This success offers hope for implementing deep learning techniques with other sorts of biological datasets where the sample size from each source is limited but combining many sources can increase the overall sample size and thus prevent overfitting while discovering domain-invariant features.

Convolutional neural network based

Convolutional neural networks (CNNs)(*38*) are great candidates for DA on data that contain spatial information such as image data. In CNNs, once features are learned using convolutional and pooling operations, the exact position of these features becomes less relevant. To address the variability of feature positions between individuals, CNNs often utilize pooling operations where the receptive field (i.e. regions in the image that the CNN's feature detector can see) is subsampled or downsampled.

Previously, many have used CCN based methods such as AlexNet(*35*) and ResNet(*180*) as the backbone in the architecture of feature extractor section in DA techniques in the field of medical imaging and computer vision(*137*, *181*). For example, Chen and colleagues(*182*) developed a novel technique using a multi-view convolutional autoencoder, which combined latent variables and searchlight-based analysis, to align fMRI data from multiple human subjects and map those subjects' data into a common space. The technique was able to preserve the spatial locality of the voxels, showing comparable or superior decoding accuracy compared to other standard techniques such as SRM or standard searchlight analysis(*182*).

Recurrent neural network based

Recurrent neural networks (RNNs) contain one or more loops in their directed connection graph through which their internal state will evolve over time in discrete steps. RNNs are often used to

process time-series data such as video or text, in which a new frame or character is fed into the network at each time step. Essentially, RNNs resemble Artificial Neural Networks (ANNs) with hidden layers that feed back onto themselves. This allows the layers to not only pass on the information to the next layer but also update their own weights and all the weights in previous layers. In other words, RNNs are many copies of the same ANNs that pass outputs to a successor. This makes RNNs great networks that can model phenomena which happen in a sequence of actions making them good candidates to extract features from times series data such as decoding emotions from EEG(*183*), movement control of prosthetics using electromyography (EMG)(*184*), or protein-protein interactions(*185*, *186*). RNNs can thus be used in DA approaches: For instance, Sonsil(*184*) and colleagues developed a method where the EMG data from one individual (source domain) was used to train a neural network to predict the gestures of another individual (target domain). They used a combination of RNN and adversarial domain adaptation (ADA)(*187*) by summing up the loss functions of the RNN predictor and the discriminator.

Discovering domain-invariant spaces: a sampling of current algorithms

Having introduced classifications and useful vocabulary both above and in the main text, we also want to direct the reader to a usefully curated corner of the DA literature. In this section, we focus on DA methods that aim to discover domain-invariant (often lower dimensional) spaces of features, as these kinds of spaces may be argued to best serve the goal of discovering generalizable truths in biology.

To discover domain-invariant spaces, we aim to find a projection matrix that minimizes statistical distance between two domains (e.g. the Maximum Mean Discrepancy (MMD)). For instance, the Distribution Matching Embedding (DME)(*188*) aims to find a projection matrix, W, that minimizes MMD such that W remains orthogonal (i.e. $W^T W = I$)). An alternative approach is to learn the common feature space using deep learning models (i.e. deep domain adaptation). However, these methods usually require a large number of samples for training. There have been some deep domain adaptation methods that have been successful on relatively smaller datasets, such as the Office-31(*47*) dataset (with ~ 132 samples per category form 3 domains), using with AlexNet(*35*) or ResNet-50(*180*) backbones(*120*, *138*, *189*, *190*). However, it is crucial to note that: (1) these are image datasets, which (2) contain substantially more samples compared to many biological datasets despite their relative sparseness compared to standard benchmark datasets. Therefore, careful consideration is necessary when applying deep DA on biological datasets.

Table S1 describes a summary of the subcategories of domain-invariant-feature based methods along with their limitations and strengths. These approaches and their kin can ideally be used, and further developed, to help discover generalizable truths in biological data which are not limited by the idiosyncrasies of a few small datasets.

**Alignment type**	**Limitations**	**Benefits**	**Sample methods / citations**
Shallow DA: discrepancy based<br><br>(e.g. MMD(*191*), correlation alignment (CORAL)(*136*), Contrastive domain discrepancy (CCD)(*192*)	Is not assumption-free, limiting its learning ability in finding domain-invariant features.	Requires relatively less amount of data.	M3SDA(*37*)<br>Guo et al.(*137*)<br>Zhu et al.(*120*)<br>Hoffman et al.(*181*)<br>Guo et al.(*193*)
Deep DA: discrepancy based	Requires relatively more samples; some metrics such as MMD and CORAL might require assumptions of kernel function for calculating the distance between distributions.	Can find highly non-linear relationships between domains and the common feature space-the alignment part do not introduce new parameters.	Long et al.(*48*)<br>Deep CORAL(*114*)<br>Contastive Domain Adaptation (CDA)(*192*)<br>Deep-JDOT(*194*)
Reconstruction based	Requires relatively more samples.	Assumption free; can find non-linear relationships between domains and the common feature space; does not need a distance metric.	DRCN(*195*)<br>DSN(*179*)<br>MTAE(*196*)
Adversarial based (e.g. GAN loss, H-divergence, Wasserstein distance(*194*))	Requires many samples, since discriminator is a network that introduces more parameters to be learned.	Effective for medical imaging data such as MRI, CT.	ADDA(*174*)<br>Tsai et al.(*197*)<br>DANN(*95*)<br>CycleGAN(*198*)<br>CyckeEmotionGAN(*199*)

Table S1. Sample algorithms used in DA which seek domain-invariant spaces, shared across two or more domains, by finding a projection that minimizes the discrepancy between domains.

REFERENCES AND NOTES

1. L. N. Ross, D. S. Bassett, Causation in neuroscience: Keeping mechanism meaningful. *Nat. Rev. Neurosci.* **25**, 81–90 (2024).

2. A. J. DeGrave, J. Janizek, S.-I. Lee, AI for radiographic COVID-19 detection selects shortcuts over signal. *Nat. Mach. Intell.* **3**, 610–619 (2021).

3. X. Li, Y. Gu, N. Dvornek, L. H. Staib, P. Ventola, J. S. Duncan, Multi-site fMRI analysis using privacy-preserving federated learning and domain adaptation: ABIDE results. *Med. Image Anal.* **65**, 101765 (2020).

4. M. Zizienová, New OSF metadata to support data sharing policy compliance. (2023).

5. M. A. Musen, C. A. Bean, K.-H. Cheung, M. Dumontier, K. A. Durante, O. Gevaert, A. Gonzalez-Beltran, P. Khatri, S. H. Kleinstein, M. J. O'Connor, Y. Pouliot, P. Rocca-Serra, S.-A. Sansone, J. A. Wiser, CEDAR team, The center for expanded data annotation and retrieval. *J. Am. Med. Inform. Assoc.* **22**, 1148–1152 (2015).

6. S. Zhao, X. Zhao, G. Ding, K. Keutzer, EmotionGAN: Unsupervised domain adaptation for learning discrete probability distributions of image emotions, in *Proceedings of the 26th ACM International Conference on Multimedia* (Association for Computing Machinery, 2018) *MM '18*, pp. 1319–1327.

7. A. Torralba, A. A. Efros, Unbiased look at dataset bias, in *CVPR 2011* (2011), pp. 1521–1528.

8. H. Kashyap, H. A. Ahmed, N. Hoque, S. Roy, D. K. Bhattacharyya, Big data analytics in bioinformatics: A machine learning perspective. arXiv:1506.05101 [cs.CE] (2015).

9. L. Duan, D. Xu, I. Tsang, Learning with augmented features for heterogeneous domain adaptation. arXiv:1206.4660 [cs.LG] (2012).

10. M. Harel, S. Mannor, Learning from multiple outlooks. arXiv:1005.0027 [cs.LG] (2010).

11. P. Prettenhofer, B. Stein, Cross-language text classification using structural correspondence learning, in *Proceedings of the 48th Annual Meeting of the Association for Computational Linguistics* (Association for Computational Linguistics, 2010), pp. 1118–1127.

12. J. Zhou, S. Pan, I. Tsang, Y. Yan, Hybrid heterogeneous transfer learning through deep learning. *AAAI* **28**, 10.1609/aaai.v28i1.8961 (2014).

13. S. J. Pan, Q. Yang, A survey on transfer learning. *IEEE Trans. Knowl. Data Eng.* **22**, 1345–1359 (2010).

14. W. Ling, J. Lu, N. Zhao, A. Lulla, A. M. Plantinga, W. Fu, A. Zhang, H. Liu, H. Song, Z. Li, J. Chen, T. W. Randolph, W. L. A. Koay, J. R. White, L. J. Launer, A. A. Fodor, K. A. Meyer, M. C. Wu, Batch effects removal for microbiome data via conditional quantile regression. *Nat. Commun.* **13**, 5418 (2022).

15. C. W. Law, Y. Chen, W. Shi, G. K. Smyth, voom: Precision weights unlock linear model analysis tools for RNA-seq read counts. *Genome Biol.* **15**, R29 (2014).

16. G. Litjens, T. Kooi, B. E. Bejnordi, A. A. A. Setio, F. Ciompi, M. Ghafoorian, J. A. W. M. van der Laak, B. van Ginneken, C. I. Sánchez, A survey on deep learning in medical image analysis. *Med. Image Anal.* **42**, 60–88 (2017).

17. Y. Zhang, Y. Wei, Q. Wu, P. Zhao, S. Niu, J. Huang, M. Tan, Collaborative unsupervised domain adaptation for medical image diagnosis. *IEEE Trans. Image Process.* **29**, 7834–7844 (2020).

18. C. Chen, Q. Dou, H. Chen, J. Qin, P.-A. Heng, Synergistic image and feature adaptation: Towards cross-modality domain adaptation for medical image segmentation. *AAAI* **33**, 865–872 (2019).

19. B. Berger, N. M. Daniels, Y. W. Yu, Computational biology in the 21st century: Scaling with compressive algorithms. *Commun. ACM* **59**, 72–80 (2016).

20. K. Lan, D.-T. Wang, S. Fong, L.-S. Liu, K. K. L. Wong, N. Dey, A survey of data mining and deep learning in bioinformatics. *J. Med. Syst.* **42**, 139 (2018).

21. K. A. Shastry, H. A. Sanjay, Machine learning for bioinformatics, in *Statistical Modelling and Machine Learning Principles for Bioinformatics Techniques, Tools, and Applications*, K. G. Srinivasa, G. M. Siddesh, S. R. Manisekhar, Eds. (Springer Singapore, 2020), pp. 25–39.

22. M. W. Libbrecht, W. S. Noble, Machine learning applications in genetics and genomics. *Nat. Rev. Genet.* **16**, 321–332 (2015).

23. P. Liu, X. Qiu, X. Huang, Adversarial multi-task learning for text classification. arXiv:1704.05742 [cs.CL] (2017).

24. A. Krizhevsky, I. Sutskever, G. E. Hinton, ImageNet classification with deep convolutional neural networks. *Commun. ACM* **60**, 84–90 (2017).

25. H. Venkateswara, J. Eusebio, S. Chakraborty, S. Panchanathan, Deep hashing network for unsupervised domain adaptation, in *Proceedings of the IEEE Conference on Computer Vision and Pattern Recognition* (2017), pp. 5018–5027.

26. X. Peng, Q. Bai, X. Xia, Z. Huang, K. Saenko, B. Wang, Moment matching for multi-source domain adaptation, in *Proceedings of the IEEE/CVF International Conference on Computer Vision* (2019), pp. 1406–1415.

27. Y. Lecun, L. Bottou, Y. Bengio, P. Haffner, Gradient-based learning applied to document recognition. *Proc. IEEE* **86**, 2278–2324 (1998).

28. A. Torralba, R. Fergus, W. T. Freeman, 80 million tiny images: A large data set for nonparametric object and scene recognition. *IEEE Trans. Pattern Anal. Mach. Intell.* **30**, 1958–1970 (2008).

29. A. Krizhevsky, G. Hinton, Others, Learning multiple layers of features from tiny images. (2009).

30. N. Altman, M. Krzywinski, The curse(s) of dimensionality. *Nat. Methods* **15**, 399–400 (2018).

31. P. D. Schloss, Identifying and overcoming threats to reproducibility, replicability, robustness, and generalizability in microbiome research. *MBio* **9**, e00525-18 (2018).

32. B. O. Turner, E. J. Paul, M. B. Miller, A. K. Barbey, Small sample sizes reduce the replicability of task-based fMRI studies. *Commun Biol* **1**, 62 (2018).

33. S. Zhou, C. R. Cox, H. Lu, Improving whole-brain neural decoding of fMRI with domain adaptation, in *Machine Learning in Medical Imaging* (Springer International Publishing, 2019), pp. 265–273.

34. M. Wasikowski, X.-W. Chen, Combating the small sample class imbalance problem using feature selection. *IEEE Trans. Knowl. Data Eng.* **22**, 1388–1400 (2010).

35. H. He, E. A. Garcia, Learning from imbalanced data. *IEEE Trans. Knowl. Data Eng.* **21**, 1263–1284 (2009).

36. K. Saenko, B. Kulis, M. Fritz, T. Darrell, Adapting visual category models to new domains. in Computer Vision–ECCV 2010 (Springer, 2010), pp. 213–226.

37. M. Long, H. Zhu, J. Wang, M. I. Jordan, Deep transfer learning with joint adaptation networks, arXiv:1605.06636 [cs.LG] (2016).

38. M. Long, Y. Cao, J. Wang, M. Jordan, Learning transferable features with deep adaptation networks, in *Proceedings of the 32nd International Conference on Machine Learning*, F. Bach, D. Blei, Eds. (PMLR, 2015) vol. 37 of *Proceedings of Machine Learning Research*, pp. 97–105.

39. E. Tzeng, J. Hoffman, N. Zhang, K. Saenko, T. Darrell, Deep domain confusion: Maximizing for domain invariance, arXiv:1412.3474 [cs.CV] (2014).

40. I. Shavitt, E. Segal, Regularization learning networks: Deep learning for tabular datasets. arXiv:1805.06440 [stat.ML] (2018).

41. S. Ö. Arik, T. Pfister, TabNet: Attentive interpretable tabular learning. *AAAI* **35**, 6679–6687 (2021).

42. D. McElfresh, S. Khandagale, J. Valverde, C. V. Prasad, G. Ramakrishnan, M. Goldblum, C. White, When do neural nets outperform boosted trees on tabular data? *Adv. Neural Inf. Process. Syst.* **36**, 76336–76369 (2023).

43. L. Grinsztajn, E. Oyallon, G. Varoquaux, Why do tree-based models still outperform deep learning on typical tabular data? in *Advances in Neural Information Processing Systems*, S. Koyejo, S. Mohamed, A. Agarwal, D. Belgrave, K. Cho, A. Oh, Eds. (Curran Associates Inc., 2022) vol. 35, pp. 507–520.

44. Y. Yang, S. Soatto, FDA: Fourier domain adaptation for semantic segmentation, in *2020 IEEE/CVF Conference on Computer Vision and Pattern Recognition (CVPR)* (IEEE, 2020), pp. 4085–4095.

45. W. Hong, Z. Wang, M. Yang, J. Yuan, Conditional generative adversarial network for structured domain adaptation, in 2018 *IEEE/CVF Conference on Computer Vision and Pattern Recognition* (IEEE, 2018), pp. 1335–1344.

46. S. Motiian, Q. Jones, S. Iranmanesh, G. Doretto, Few-shot adversarial domain adaptation. *Adv. Neural Inf. Process. Syst.* **30**, (2017).

47. K. Sohn, S. Liu, G. Zhong, X. Yu, M.-H. Yang, M. Chandraker, Unsupervised domain adaptation for face recognition in unlabeled videos, arXiv:1708.02191 [cs.CV] (2017).

48. B. Ghosh-Dastidar, J. L. Schafer, Multiple edit/multiple imputation for multivariate continuous data. *J. Am. Stat. Assoc.* **98**, 807–817 (2003).

49. N. Eisemann, A. Waldmann, A. Katalinic, Imputation of missing values of tumour stage in population-based cancer registration. *BMC Med. Res. Methodol.* **11**, 129 (2011).

50. D. van Dijk, J. Nainys, R. Sharma, P. Kaithail, A. J. Carr, K. R. Moon, L. Mazutis, G. Wolf, S. Krishnaswamy, D. Pe'er, MAGIC: A diffusion-based imputation method reveals gene-gene interactions in single-cell RNA-sequencing data. bioRxiv 111591 [Preprint] (2017). https://doi.org/10.1101/111591.

51. M. Zitnik, F. Nguyen, B. Wang, J. Leskovec, A. Goldenberg, M. M. Hoffman, Machine learning for integrating data in biology and medicine: Principles, practice, and opportunities. *Inf. Fusion* **50**, 71–91 (2019).

52. J. V. Haxby, J. S. Guntupalli, A. C. Connolly, Y. O. Halchenko, B. R. Conroy, M. I. Gobbini, M. Hanke, P. J. Ramadge, A common, high-dimensional model of the representational space in human ventral temporal cortex. *Neuron* **72**, 404–416 (2011).

53. C. Xu, S. A. Jackson, Machine learning and complex biological data. *Genome Biol.* **20**, 76 (2019).

54. V. Z. Marmarelis, Identification of nonlinear biological systems using Laguerre expansions of kernels. *Ann. Biomed. Eng.* **21**, 573–589 (1993).

55. D. Singh, H. Climente-Gonzalez, M. Petrovich, E. Kawakami, M. Yamada, FsNet: Feature selection network on high-dimensional biological data, in *2023 International Joint Conference on Neural Networks (IJCNN)* (IEEE, 2023), pp. 1–9.

56. A. Y. Pan, Statistical analysis of microbiome data: The challenge of sparsity. *Curr. Opin. Endocr. Metab. Res.* **19**, 35–40 (2021).

57. T. Zhou, M. Liu, K.-H. Thung, D. Shen, Latent representation learning for Alzheimer's disease diagnosis with incomplete multi-modality neuroimaging and genetic data. *IEEE Trans. Med. Imaging* **38**, 2411–2422 (2019).

58. P. Samartsidis, S. Montagna, A. R. Laird, P. T. Fox, T. D. Johnson, T. E. Nichols, Estimating the prevalence of missing experiments in a neuroimaging meta-analysis. *Res. Synth. Methods* **11**, 866–883 (2020).

59. T. Zhou, K.-H. Thung, M. Liu, F. Shi, C. Zhang, D. Shen, Multi-modal latent space inducing ensemble SVM classifier for early dementia diagnosis with neuroimaging data. *Med. Image Anal.* **60**, 101630 (2020).

60. E. L. Busch, L. Slipski, M. Feilong, J. S. Guntupalli, M. V. di Oleggio Castello, J. F. Huckins, S. A. Nastase, M. I. Gobbini, T. D. Wager, J. V. Haxby, Hybrid Hyperalignment: A single

high-dimensional model of shared information embedded in cortical patterns of response and functional connectivity. *Neuroimage* **233**, 117975 (2021).

61. P. Wei, Y. Ke, C. K. Goh, A general domain specific feature transfer framework for hybrid domain adaptation. *IEEE Trans. Knowl. Data Eng.* **31**, 1440–1451 (2019).

62. C. Wang, S. Mahadevan, Heterogeneous domain adaptation using manifold alignment, in *Twenty-Second International Joint Conference on Artificial Intelligence* (2011), pp. 1541–1546. https://aaai.org/ocs/index.php/IJCAI/IJCAI11/paper/viewPaper/3207.

63. B. J. Callahan, P. J. McMurdie, M. J. Rosen, A. W. Han, A. J. A. Johnson, S. P. Holmes, DADA2: High-resolution sample inference from Illumina amplicon data. *Nat. Methods* **13**, 581–583 (2016).

64. A. Amir, D. McDonald, J. A. Navas-Molina, E. Kopylova, J. T. Morton, Z. Zech Xu, E. P. Kightley, L. R. Thompson, E. R. Hyde, A. Gonzalez, R. Knight, Deblur rapidly resolves single-nucleotide community sequence patterns. *mSystems* **2**, e00191-16 (2017).

65. O. Esteban, C. J. Markiewicz, R. W. Blair, C. A. Moodie, A. I. Isik, A. Erramuzpe, J. D. Kent, M. Goncalves, E. DuPre, M. Snyder, H. Oya, S. S. Ghosh, J. Wright, J. Durnez, R. A. Poldrack, K. J. Gorgolewski, fMRIPrep: A robust preprocessing pipeline for functional MRI. *Nat. Methods* **16**, 111–116 (2019).

66. R. W. Cox, AFNI: Software for analysis and visualization of functional magnetic resonance neuroimages. *Comput. Biomed. Res.* **29**, 162–173 (1996).

67. R. W. Cox, J. S. Hyde, Software tools for analysis and visualization of fMRI data. *NMR Biomed.* **10**, 171–178 (1997).

68. M. W. Woolrich, S. Jbabdi, B. Patenaude, M. Chappell, S. Makni, T. Behrens, C. Beckmann, M. Jenkinson, S. M. Smith, Bayesian analysis of neuroimaging data in FSL. *Neuroimage* **45**, S173–S186 (2009).

69. S. M. Smith, M. Jenkinson, M. W. Woolrich, C. F. Beckmann, T. E. J. Behrens, H. Johansen-Berg, P. R. Bannister, M. De Luca, I. Drobnjak, D. E. Flitney, R. K. Niazy, J. Saunders, J.

Vickers, Y. Zhang, N. De Stefano, J. M. Brady, P. M. Matthews, Advances in functional and structural MR image analysis and implementation as FSL. *Neuroimage* **23**, S208–S219 (2004).

70. M. Jenkinson, C. F. Beckmann, T. E. J. Behrens, M. W. Woolrich, S. M. Smith, FSL. *Neuroimage* **62**, 782–790 (2012).

71. A. Andronache, C. Rosazza, D. Sattin, M. Leonardi, L. D'Incerti, L. Minati, Coma Research Centre (CRC)–Besta Institute, Impact of functional MRI data preprocessing pipeline on default-mode network detectability in patients with disorders of consciousness. *Front. Neuroinform.* **7**, 16 (2013).

72. N. Bhagwat, A. Barry, E. W. Dickie, S. T. Brown, G. A. Devenyi, K. Hatano, E. DuPre, A. Dagher, M. Chakravarty, C. M. T. Greenwood, B. Misic, D. N. Kennedy, J.-B. Poline, Understanding the impact of preprocessing pipelines on neuroimaging cortical surface analyses. *Gigascience* **10**, giaa155 (2021).

73. M. A. Lindquist, S. Geuter, T. D. Wager, B. S. Caffo, Modular preprocessing pipelines can reintroduce artifacts into fMRI data. *Hum. Brain Mapp.* **40**, 2358–2376 (2019).

74. E. Ibrahimi, M. B. Lopes, X. Dhamo, A. Simeon, R. Shigdel, K. Hron, B. Stres, D. D'Elia, M. Berland, L. J. Marcos-Zambrano, Overview of data preprocessing for machine learning applications in human microbiome research. *Front. Microbiol.* **14**, 1250909 (2023).

75. G. Cammarota, G. Ianiro, A. Ahern, C. Carbone, A. Temko, M. J. Claesson, A. Gasbarrini, G. Tortora, Gut microbiome, big data and machine learning to promote precision medicine for cancer. *Nat. Rev. Gastroenterol. Hepatol.* **17**, 635–648 (2020).

76. Y.-M. Kim, J.-B. Poline, G. Dumas, Experimenting with reproducibility: A case study of robustness in bioinformatics. *Gigascience* **7**, (2018).

77. X. Liu, J. Wu, W. Li, Q. Liu, L. Tian, H. Huang, Domain adaptation via low rank and class discriminative representation for autism spectrum disorder identification: A multi-site fMRI study. *IEEE Trans. Neural Syst. Rehabil. Eng.* **31**, 806–817 (2023).

78. M. Pominova, E. Kondrateva, M. Sharaev, A. Bernstein, E. Burnaev, Fader networks for domain adaptation on fMRI: ABIDE-II study, in *Thirteenth International Conference on Machine Vision* (SPIE, 2021) vol. 11605, pp. 570–577.

79. X. Liu, H. Huang, Alterations of functional connectivities associated with autism spectrum disorder symptom severity: A multi-site study using multivariate pattern analysis. *Sci. Rep.* **10**, 4330 (2020).

80. A. van Opbroek, M. A. Ikram, M. W. Vernooij, M. de Bruijne, Transfer learning improves supervised image segmentation across imaging protocols. *IEEE Trans. Med. Imaging* **34**, 1018–1030 (2015).

81. W. M. Kouw, M. Loog, L. W. Bartels, A. M. Mendrik, MR acquisition-invariant representation learning, arXiv:1709.07944 [cs.CV] (2017).

82. B. Wang, F. Sun, Y. Luan, Comparison of the effectiveness of different normalization methods for metagenomic cross-study phenotype prediction under heterogeneity. *Sci. Rep.* **14**, 7024 (2024).

83. X. Yang, C. Deng, T. Liu, D. Tao, Heterogeneous graph attention network for unsupervised multiple-target domain adaptation. *IEEE Trans. Pattern Anal. Mach. Intell.* **44**, 1992–2003 (2022).

84. F. Liu, G. Zhang, J. Lu, Heterogeneous domain adaptation: An unsupervised approach. *IEEE Trans. Neural Netw. Learn. Syst.* **31**, 5588–5602 (2020).

85. Y. Ganin, E. Ustinova, H. Ajakan, P. Germain, H. Larochelle, F. Laviolette, M. Marchand, V. Lempitsky, Domain-adversarial training of neural networks, in *Domain Adaptation in Computer Vision Applications* (Springer International Publishing, 2017) *Advances in computer vision and pattern recognition*, pp. 189–209.

86. R. Vinuesa, B. Sirmacek, Interpretable deep-learning models to help achieve the sustainable development goals. *Nat. Mach. Intell.* **3**, 926–926 (2021).

87. D. Arpit, S. Jastrzębski, N. Ballas, D. Krueger, E. Bengio, M. S. Kanwal, T. Maharaj, A. Fischer, A. Courville, Y. Bengio, S. Lacoste-Julien, A closer look at memorization in deep networks, in *Proceedings of the 34th International Conference on Machine Learning*, D. Precup, Y. W. Teh, Eds. (PMLR, 06--11 Aug. 2017) vol. 70 of *Proceedings of Machine Learning Research*, pp. 233–242.

88. P. W. Koh, P. Liang, Understanding Black-box Predictions via Influence Functions, in *Proceedings of the 34th International Conference on Machine Learning*, D. Precup, Y. W. Teh, Eds. (PMLR, 06--11 Aug 2017) vol. 70 of *Proceedings of Machine Learning Research*, pp. 1885–1894.

89. A. Mehra, B. Kailkhura, P.-Y. Chen, J. Hamm, Understanding the limits of unsupervised domain adaptation via data poisoning. *Adv. Neural Inf. Process. Syst.* **1327**, 17347–17359 (2021).

90. S. Ben-David, T. Lu, T. Luu, D. Pál, Impossibility theorems for domain adaptation. *AISTATS* **9**, 129–136 (2010).

91. I. Redko, A. Habrard, M. Sebban, On the analysis of adaptability in multi-source domain adaptation. *Mach. Learn.* **108**, 1635–1652 (2019).

92. H. Liu, M. Long, J. Wang, M. Jordan, Transferable adversarial training: A general approach to adapting deep classifiers, in *Proceedings of the 36th International Conference on Machine Learning*, K. Chaudhuri, R. Salakhutdinov, Eds. (PMLR, 09–15 Jun 2019) vol. 97 of *Proceedings of Machine Learning Research*, pp. 4013–4022.

93. Z. Wang, Z. Dai, B. Póczos, J. Carbonell, Characterizing and avoiding negative transfer, in *2019 IEEE/CVF Conference on Computer Vision and Pattern Recognition (CVPR)* (IEEE, 2019), pp. 11285–11294.

94. H. Ajakan, P. Germain, H. Larochelle, F. Laviolette, M. Marchand, Domain-adversarial neural networks. arXiv:1412.4446 [stat.ML] (2014).

95. Y. S. Chan, H. T. Ng, Word sense disambiguation with distribution estimation. https://ijcai.org/Proceedings/05/Papers/1543.pdf.

96. W. M. Kouw, M. Loog, An introduction to domain adaptation and transfer learning. arXiv:1812.11806 [cs.LG] (2018).

97. M. Wang, W. Deng, Deep visual domain adaptation: A survey. *Neurocomputing* **312**, 135–153 (2018).

98. G. Wilson, D. J. Cook, A survey of unsupervised deep domain adaptation. *ACM Trans. Intell. Syst. Technol.* **11**, 1–46 (2020).

99. G. Csurka, Domain adaptation for visual applications: A comprehensive survey. arXiv:1702.05374 [cs.CV] (2017).

100. X. Liu, C. Yoo, F. Xing, H. Oh, G. El Fakhri, J.-W. Kang, J. Woo, Deep unsupervised domain adaptation: A review of recent advances and perspectives. *APSIPA Transactions on Signal and Information Processing* **11**, (2022).

101. S. Zhao, B. Li, C. Reed, P. Xu, K. Keutzer, Multi-source domain adaptation in the deep learning era: A systematic survey. arXiv:2002.12169 [cs.LG] (2020).

102. B. Fernando, A. Habrard, M. Sebban, T. Tuytelaars, Unsupervised visual domain adaptation using subspace alignment, in *Proceedings of the IEEE International Conference on Computer Vision* (2013), pp. 2960–2967.

103. B. Gong, Y. Shi, F. Sha, K. Grauman, Geodesic flow kernel for unsupervised domain adaptation, in *2012 IEEE Conference on Computer Vision and Pattern Recognition* (ieeexplore.ieee.org, 2012), pp. 2066–2073.

104. B. Sun, K. Saenko, Deep CORAL: Correlation alignment for deep domain adaptation, in *Computer Vision–ECCV 2016 Workshops* (Springer International Publishing, 2016), pp. 443–450.

105. S. Sun, H. Shi, Y. Wu, A survey of multi-source domain adaptation. *Inf. Fusion* **24**, 84–92 (2015).

106. H. S. Bhatt, A. Rajkumar, S. Roy, Multi-source iterative adaptation for cross-domain classification, in *Proceedings of the Twenty-Fifth International Joint Conference on Artificial Intelligence (IJCAI-16*), pp. 3691–3697. https://ijcai.org/Proceedings/16/Papers/519.pdf.

107. T. Matsuura, T. Harada, Domain generalization using a mixture of multiple latent domains. *AAAI* **34**, 11749–11756 (2020).

108. E. F. Montesuma, F. M. N. Mboula, Wasserstein barycenter for multi-source domain adaptation, in *2021 IEEE/CVF Conference on Computer Vision and Pattern Recognition (CVPR)* (IEEE, 2021), pp. 16785–16793.

109. Y. Mansour, M. Mohri, A. Rostamizadeh, Domain adaptation with multiple sources. *Adv. Neural Inf. Process. Syst.* **21**, 1041–1048 (2008).

110. Y. Zhu, F. Zhuang, D. Wang, Aligning domain-specific distribution and classifier for cross-domain classification from multiple sources. *AAAI* **33**, 5989–5996 (2019).

111. R. Xu, Z. Chen, W. Zuo, J. Yan, L. Lin, Deep cocktail network: Multi-source unsupervised domain adaptation with category shift, in *Proceedings of the IEEE Conference on Computer Vision and Pattern Recognition* (2018), pp. 3964–3973.

112. H. Zhao, S. Zhang, G. Wu, J. M. F. Moura, J. P. Costeira, G. J. Gordon, Adversarial multiple source domain adaptation. *Adv. Neural Inf. Process. Syst.* **31**, 8559–8570 (2018).

113. H. Guan, M. Liu, Domain adaptation for medical image analysis: A survey. *I.E.E.E. Trans. Biomed. Eng.* **69**, 1173–1185 (2022).

114. H. Daumé III, Frustratingly easy domain adaptation. arXiv:0907.1815 [cs.LG] (2009).

115. K. Saito, Y. Ushiku, T. Harada, Asymmetric tri-training for unsupervised domain adaptation, in *Proceedings of the 34th International Conference on Machine Learning*, D. Precup, Y. W.

Teh, Eds. (PMLR, 06--11 Aug 2017) vol. 70 of *Proceedings of Machine Learning Research*, pp. 2988–2997.

116. A. Shrivastava, T. Pfister, O. Tuzel, J. Susskind, W. Wang, R. Webb, Learning from simulated and unsupervised images through adversarial training. arXiv:1612.07828 [cs. CV] (2016).

117. J. Zhuo, S. Wang, W. Zhang, Q. Huang, Deep unsupervised convolutional domain adaptation, in *Proceedings of the 25th ACM International Conference on Multimedia* (Association for Computing Machinery, 2017) *MM '17*, pp. 261–269.

118. W. Li, L. Duan, X. Dong, I. W. Tsang, Learning with augmented features for supervised and semi-supervised heterogeneous domain adaptation. *IEEE Trans. Pattern Anal. Mach. Intell.* **36**, 1134–1148 (2014).

119. E. Tzeng, J. Hoffman, T. Darrell, K. Saenko, Simultaneous deep transfer across domains and tasks. arXiv:1510.02192 [cs.CV] (2015).

120. K. Saito, D. Kim, S. Sclaroff, T. Darrell, K. Saenko, Semi-supervised domain adaptation via minimax entropy, in *2019 IEEE/CVF International Conference on Computer Vision (ICCV)* (IEEE, 2019), pp. 8050–8058.

121. H. Shimodaira, Improving predictive inference under covariate shift by weighting the log-likelihood function. *J. Stat. Plan. Inference* **90**, 227–244 (2000).

122. J. Yang, R. Yan, A. G. Hauptmann, Cross-domain video concept detection using adaptive svms, in *Proceedings of the 15th ACM International Conference on Multimedia* (Association for Computing Machinery, 2007) *MM '07*, pp. 188–197.

123. L. Duan, I. W. Tsang, D. Xu, T.-S. Chua, Domain adaptation from multiple sources via auxiliary classifiers, in *Proceedings of the 26th Annual International Conference on Machine Learning* (Association for Computing Machinery, 2009) *ICML '09*, pp. 289–296.

124. L. Duan, D. Xu, S.-F. Chang, Exploiting web images for event recognition in consumer videos: A multiple source domain adaptation approach, in *2012 IEEE Conference on Computer Vision and Pattern Recognition* (2012), pp. 1338–1345.

125. J. T. Zhou, I. W.Tsang, S. J. Pan, M. Tan, Heterogeneous domain adaptation for multiple classes, in *Proceedings of the Seventeenth International Conference on Artificial Intelligence and Statistics*, S. Kaski, J. Corander, Eds. (PMLR, 2014) vol. 33 of *Proceedings of Machine Learning Research*, pp. 1095–1103.

126. B. Sun, J. Feng, K. Saenko, Return of frustratingly easy domain adaptation. *AAAI* **30**, (2016).

127. J. Guo, D. Shah, R. Barzilay, Multi-source domain adaptation with mixture of experts. arXiv:1809.02256 [cs.CL] (218).

128. M. Mancini, L. Porzi, S. R. Bulo, B. Caputo, E. Ricci, Boosting domain adaptation by discovering latent domains, in *2018 IEEE/CVF Conference on Computer Vision and Pattern Recognition* (IEEE, 2018), pp. 3771–3780.

129. S. Zhao, G. Wang, S. Zhang, Y. Gu, Y. Li, Z. Song, P. Xu, R. Hu, H. Chai, K. Keutzer, Multi-source distilling domain adaptation. *AAAI* **34**, 12975–12983 (2020).

130. M. Wang, D. Zhang, J. Huang, P.-T. Yap, D. Shen, M. Liu, Identifying autism spectrum disorder with multi-site fMRI via low-rank domain adaptation. *IEEE Trans. Med. Imaging* **39**, 644–655 (2020).

131. A. Mensch, J. Mairal, D. Bzdok, B. Thirion, G. Varoquaux, Learning neural representations of human cognition across many fMRI studies. arXiv:1710.11438 [stat.ML] (2017).

132. H. Zhang, P.-H. Chen, P. Ramadge, Transfer learning on fMRI datasets, in *Proceedings of the Twenty-First International Conference on Artificial Intelligence and Statistics*, A. Storkey, F. Perez-Cruz, Eds. (PMLR, 2018) vol. 84 of *Proceedings of Machine Learning Research*, pp. 595–603.

133. C. Huang, C. Gin, J. Fettweis, B. Foxman, B. Gelaye, D. A. MacIntyre, A. Subramaniam, W. Fraser, N. Tabatabaei, B. Callahan, Meta-analysis reveals the vaginal microbiome is a better predictor of earlier than later preterm birth. *BMC Biol.* **21**, 199 (2023).

134. J. L. Golob, T. T. Oskotsky, A. S. Tang, A. Roldan, V. Chung, C. W. Y. Ha, R. J. Wong, K. J. Flynn, A. Parraga-Leo, C. Wibrand, S. S. Minot, B. Oskotsky, G. Andreoletti, I. Kosti, J. Bletz, A. Nelson, J. Gao, Z. Wei, G. Chen, Z.-Z. Tang, P. Novielli, D. Romano, E. Pantaleo, N. Amoroso, A. Monaco, M. Vacca, M. De Angelis, R. Bellotti, S. Tangaro, A. Kuntzleman, I. Bigcraft, S. Techtmann, D. Bae, E. Kim, J. Jeon, S. Joe, Preterm Birth DREAM Community, K. R. Theis, S. Ng, Y. S. Lee, P. Diaz-Gimeno, P. R. Bennett, D. A. MacIntyre, G. Stolovitzky, S. V. Lynch, J. Albrecht, N. Gomez-Lopez, R. Romero, D. K. Stevenson, N. Aghaeepour, A. L. Tarca, J. C. Costello, M. Sirota, Microbiome preterm birth DREAM challenge: Crowdsourcing machine learning approaches to advance preterm birth research. *Cell Rep. Med.* **5**, 101350 (2024).

135. G. I. Austin, A. B. Kav, H. Park, J. Biermann, A.-C. Uhlemann, T. Korem, Processing-bias correction with DEBIAS-M improves cross-study generalization of microbiome-based prediction models. bioRxiv 579716 [Preprint] (2024). https://doi.org/10.1101/2024.02.09.579716.

136. S. Mourragui, M. Loog, M. A. van de Wiel, M. J. T. Reinders, L. F. A. Wessels, PRECISE: A domain adaptation approach to transfer predictors of drug response from pre-clinical models to tumors. *Bioinformatics* **35**, i510–i519 (2019).

137. H. Sharifi-Noghabi, S. Peng, O. Zolotareva, C. C. Collins, M. Ester, AITL: Adversarial inductive transfer learning with input and output space adaptation for pharmacogenomics. *Bioinformatics* **36**, i380–i388 (2020).

138. L. Handl, A. Jalali, M. Scherer, R. Eggeling, N. Pfeifer, Weighted elastic net for unsupervised domain adaptation with application to age prediction from DNA methylation data. *Bioinformatics* **35**, i154–i163 (2019).

139. A. Di Martino, C.-G. Yan, Q. Li, E. Denio, F. X. Castellanos, K. Alaerts, J. S. Anderson, M. Assaf, S. Y. Bookheimer, M. Dapretto, B. Deen, S. Delmonte, I. Dinstein, B. Ertl-Wagner,

D. A. Fair, L. Gallagher, D. P. Kennedy, C. L. Keown, C. Keysers, J. E. Lainhart, C. Lord, B. Luna, V. Menon, N. J. Minshew, C. S. Monk, S. Mueller, R.-A. Müller, M. B. Nebel, J. T. Nigg, K. O'Hearn, K. A. Pelphrey, S. J. Peltier, J. D. Rudie, S. Sunaert, M. Thioux, J. M. Tyszka, L. Q. Uddin, J. S. Verhoeven, N. Wenderoth, J. L. Wiggins, S. H. Mostofsky, M. P. Milham, The autism brain imaging data exchange: Towards a large-scale evaluation of the intrinsic brain architecture in autism. *Mol. Psychiatry* **19**, 659–667 (2014).

140. S. Masoudnia, R. Ebrahimpour, Mixture of experts: A literature survey. *Artif. Intell. Rev.* **42**, 275–293 (2014).

141. N. Shazeer, A. Mirhoseini, K. Maziarz, A. Davis, Q. Le, G. Hinton, J. Dean, Outrageously large neural networks: The sparsely-gated mixture-of-experts layer. arXiv:1701.06538 [cs. LG] (2017).

142. Y. Gao, Y. Zhang, Z. Cao, X. Guo, J. Zhang, Decoding brain states from fMRI signals by using unsupervised domain adaptation. *IEEE J. Biomed. Health Inform.* **24**, 1677–1685 (2020).

143. D. C. Van Essen, K. Ugurbil, E. Auerbach, D. Barch, T. E. J. Behrens, R. Bucholz, A. Chang, L. Chen, M. Corbetta, S. W. Curtiss, S. Della Penna, D. Feinberg, M. F. Glasser, N. Harel, A. C. Heath, L. Larson-Prior, D. Marcus, G. Michalareas, S. Moeller, R. Oostenveld, S. E. Petersen, F. Prior, B. L. Schlaggar, S. M. Smith, A. Z. Snyder, J. Xu, E. Yacoub, WU-Minn HCP Consortium, The human connectome project: A data acquisition perspective. *Neuroimage* **62**, 2222–2231 (2012).

144. T. Miller, Explanation in artificial intelligence: Insights from the social sciences. *Artif Intell* **267**, 1–38 (2019).

145. S. Orouji, V. Taschereau-Dumouchel, A. Cortese, Task-relevant autoencoding enhances machine learning for human neuroscience. arXiv:2208.08478 [q-bio.NC] (2022).

146. L. Hou, Regularizing label-augmented generative adversarial networks under limited data. *IEEE Access* **11**, 28966–28976 (2023).

147. R. Webster, J. Rabin, L. Simon, F. Jurie, Detecting overfitting of deep generative networks via latent recovery. arXiv:1901.03396 [cs.LG] (2019).

148. A. Gonzalez, J. A. Navas-Molina, T. Kosciolek, D. McDonald, Y. Vázquez-Baeza, G. Ackermann, J. DeReus, S. Janssen, A. D. Swafford, S. B. Orchanian, J. G. Sanders, J. Shorenstein, H. Holste, S. Petrus, A. Robbins-Pianka, C. J. Brislawn, M. Wang, J. R. Rideout, E. Bolyen, M. Dillon, J. G. Caporaso, P. C. Dorrestein, R. Knight, Qiita: Rapid, web-enabled microbiome meta-analysis. *Nat. Methods* **15**, 796–798 (2018).

149. E. Pasolli, L. Schiffer, P. Manghi, A. Renson, V. Obenchain, D. T. Truong, F. Beghini, F. Malik, M. Ramos, J. B. Dowd, C. Huttenhower, M. Morgan, N. Segata, L. Waldron, Accessible, curated metagenomic data through ExperimentHub. *Nat. Methods* **14**, 1023–1024 (2017).

150. A. Amir, E. Ozel, Y. Haberman, N. Shental, Achieving pan-microbiome biological insights via the dbBact knowledge base. *Nucleic Acids Res.* **51**, 6593–6608 (2023).

151. R. J. Abdill, S. P. Graham, V. Rubinetti, F. W. Albert, C. S. Greene, S. Davis, R. Blekhman, Integration of 168,000 samples reveals global patterns of the human gut microbiome. bioRxiv 560955 [Preprint] (2023). https://doi.org/10.1101/2023.10.11.560955.

152. K. Muandet, D. Balduzzi, B. Schölkopf, Domain generalization via invariant feature representation. *Proc. Mach. Learn.* **28**, 10–18 (2013).

153. J. T. Leek, R. B. Scharpf, H. C. Bravo, D. Simcha, B. Langmead, W. E. Johnson, D. Geman, K. Baggerly, R. A. Irizarry, Tackling the widespread and critical impact of batch effects in high-throughput data. *Nat. Rev. Genet.* **11**, 733–739 (2010).

154. P. I. Costea, G. Zeller, S. Sunagawa, E. Pelletier, A. Alberti, F. Levenez, M. Tramontano, M. Driessen, R. Hercog, F.-E. Jung, J. R. Kultima, M. R. Hayward, L. P. Coelho, E. Allen-Vercoe, L. Bertrand, M. Blaut, J. R. M. Brown, T. Carton, S. Cools-Portier, M. Daigneault, M. Derrien, A. Druesne, W. M. de Vos, B. B. Finlay, H. J. Flint, F. Guarner, M. Hattori, H. Heilig, R. A. Luna, J. van Hylckama Vlieg, J. Junick, I. Klymiuk, P. Langella, E. Le Chatelier, V. Mai, C. Manichanh, J. C. Martin, C. Mery, H. Morita, P. W. O'Toole, C.

Orvain, K. R. Patil, J. Penders, S. Persson, N. Pons, M. Popova, A. Salonen, D. Saulnier, K. P. Scott, B. Singh, K. Slezak, P. Veiga, J. Versalovic, L. Zhao, E. G. Zoetendal, S. D. Ehrlich, J. Dore, P. Bork, Towards standards for human fecal sample processing in metagenomic studies. *Nat. Biotechnol.* **35**, 1069–1076 (2017).

155. N. Herndon, D. Caragea, Naıve bayes domain adaptation for biological sequences, in *Proceedings of the 4th International Conference on Bioinformatics Models, Methods and Algorithms, BIOINFORMATICS* (2013), pp. 62–70.

156. J. Wirbel, P. T. Pyl, E. Kartal, K. Zych, A. Kashani, A. Milanese, J. S. Fleck, A. Y. Voigt, A. Palleja, R. Ponnudurai, S. Sunagawa, L. P. Coelho, P. Schrotz-King, E. Vogtmann, N. Habermann, E. Niméus, A. M. Thomas, P. Manghi, S. Gandini, D. Serrano, S. Mizutani, H. Shiroma, S. Shiba, T. Shibata, S. Yachida, T. Yamada, L. Waldron, A. Naccarati, N. Segata, R. Sinha, C. M. Ulrich, H. Brenner, M. Arumugam, P. Bork, G. Zeller, Meta-analysis of fecal metagenomes reveals global microbial signatures that are specific for colorectal cancer. *Nat. Med.* **25**, 679–689 (2019).

157. A. M. Thomas, P. Manghi, F. Asnicar, E. Pasolli, F. Armanini, M. Zolfo, F. Beghini, S. Manara, N. Karcher, C. Pozzi, S. Gandini, D. Serrano, S. Tarallo, A. Francavilla, G. Gallo, M. Trompetto, G. Ferrero, S. Mizutani, H. Shiroma, S. Shiba, T. Shibata, S. Yachida, T. Yamada, J. Wirbel, P. Schrotz-King, C. M. Ulrich, H. Brenner, M. Arumugam, P. Bork, G. Zeller, F. Cordero, E. Dias-Neto, J. C. Setubal, A. Tett, B. Pardini, M. Rescigno, L. Waldron, A. Naccarati, N. Segata, Metagenomic analysis of colorectal cancer datasets identifies cross-cohort microbial diagnostic signatures and a link with choline degradation. *Nat. Med.* **25**, 667–678 (2019).

158. H. Daumé III, A. Kumar, A. Saha, Frustratingly easy semi-supervised domain adaptation, in *Proceedings of the 2010 Workshop on Domain Adaptation for Natural Language Processing* (2010), pp. 53–59.

159. M. Schneider, L. Wang, C. Marr, Evaluation of domain adaptation approaches for robust classification of heterogeneous biological data sets, in *Artificial Neural Networks and Machine Learning–ICANN 2019: Deep Learning* (Springer International Publishing, 2019), pp. 673–686.

160. J. Blitzer, R. McDonald, F. Pereira, Domain adaptation with structural correspondence learning, in *Proceedings of the 2006 Conference on Empirical Methods in Natural Language Processing* (2006), pp. 120–128.

161. M. Baktashmotlagh, M. T. Harandi, B. C. Lovell, M. Salzmann, Domain adaptation on the statistical manifold, in *Proceedings of the IEEE Conference on Computer Vision and Pattern Recognition* (2014), pp. 2481–2488.

162. B. Gong, K. Grauman, F. Sha, Connecting the dots with landmarks: Discriminatively learning domain-invariant features for unsupervised domain adaptation, in *Proceedings of the 30th International Conference on Machine Learning*, S. Dasgupta, D. McAllester, Eds. (PMLR, 2013) vol. 28 of *Proceedings of Machine Learning Research*, pp. 222–230.

163. H. Hotelling, Relations between two sets of variates. *Biometrika* **28**, 321–377 (1936).

164. D. R. Hardoon, S. Szedmak, J. Shawe-Taylor, Canonical correlation analysis: An overview with application to learning methods. *Neural Comput.* **16**, 2639–2664 (2004).

165. Y.-R. Yeh, C.-H. Huang, Y.-C. F. Wang, Heterogeneous domain adaptation and classification by exploiting the correlation subspace. *IEEE Trans. Image Process.* **23**, 2009–2018 (2014).

166. P. L. Lai, C. Fyfe, Kernel and nonlinear canonical correlation analysis. *Int. J. Neural Syst.* **10**, 365–377 (2000).

167. F. R. Bach, Kernel independent component analysis. *J. Mach. Learn. Res.* **3**, 1–48 (2002).

168. J. V. Haxby, J. S. Guntupalli, S. A. Nastase, M. Feilong, Hyperalignment: Modeling shared information encoded in idiosyncratic cortical topographies. *eLife* **9**, e56601 (2020).

169. J. S. Guntupalli, M. Feilong, J. V. Haxby, A computational model of shared fine-scale structure in the human connectome. *PLOS Comput. Biol.* **14**, e1006120 (2018).

170. C. Wang, S. Mahadevan, Manifold alignment without correspondence, in *Twenty-First International Joint Conference on Artificial Intelligence* (2009; https://aaai.org/ocs/index.php/IJCAI/IJCAI-09/paper/viewPaper/446).

171. P.-H. C. Chen, J. Chen, Y. Yeshurun, U. Hasson, J. Haxby, P. J. Ramadge, A reduced-fimension fMRI shared response ,odel, in *Advances in Neural Information Processing Systems*, C. Cortes, N. Lawrence, D. Lee, M. Sugiyama, R. Garnett, Eds. (Curran Associates Inc., 2015) vol. 28, pp. 460–468.

172. M. R. McLaren, A. D. Willis, B. J. Callahan, Consistent and correctable bias in metagenomic sequencing experiments. *eLife* **8**, e46923 (2019).

173. H. Wang, W. Yang, Z. Lin, Y. Yu, TMDA: Task-specific multi-source domain adaptation via clustering embedded adversarial training, in *2019 IEEE International Conference on Data Mining (ICDM)* (2019), pp. 1372–1377.

174. E. Tzeng, J. Hoffman, K. Saenko, T. Darrell, Adversarial discriminative domain adaptation, in *Proceedings of the IEEE Conference on Computer Vision and Pattern Recognition* (openaccess.thecvf.com, 2017), pp. 7167–7176.

175. M.-Y. Liu, O. Tuzel, Coupled Generative Adversarial Networks, in *Advances in Neural Information Processing Systems*, D. Lee, M. Sugiyama, U. Luxburg, I. Guyon, R. Garnett, Eds. (Curran Associates Inc., 2016; https://proceedings.neurips.cc/paper/2016/file/502e4a16930e414107ee22b6198c578f-Paper.pdf), vol. 29.

176. I. Goodfellow, J. Pouget-Abadie, M. Mirza, B. Xu, D. Warde-Farley, S. Ozair, A. Courville, Y. Bengio, Generative adversarial nets, in *Advances in Neural Information Processing Systems*, Z. Ghahramani, M. Welling, C. Cortes, N. Lawrence, K. Q. Weinberger, Eds. (Curran Associates, Inc., 2014; https://proceedings.neurips.cc/paper/2014/file/5ca3e9b122f61f8f06494c97b1afccf3-Paper.pdf), vol. 27.

177. Y. Bengio, Learning deep architectures for AI. *Found. Trends Mach. Learn.* **2**, 1–127 (2009).

178. P. Vincent, Stacked denoising autoencoders: Learning useful representations in a deep network with a local denoising criterion. *J. Mach. Learn. Res.* **11**, 3371–3408 (2010).

179. K. Bousmalis, G. Trigeorgis, N. Silberman, D. Krishnan, D. Erhan, Domain separation networks, in *Advances in Neural Information Processing Systems*. D. Lee, M. Sugiyama, U. Luxburg, I. Guyon, R. Garnett, Eds. (Curran Associates Inc., 2016; https://proceedings.neurips.cc/paper/2016/file/45fbc6d3e05ebd93369ce542e8f2322d-Paper.pdf) vol. 29.

180. K. He, X. Zhang, S. Ren, J. Sun, Deep residual learning for image recognition, in *Proceedings of the IEEE Conference on Computer Vision and Pattern Recognition* (2016), pp. 770–778.

181. J. Hoffman, M. Mohri, N. Zhang, Algorithms and theory for multiple-source adaptation. arXiv:1805.08727 [cs.LG] (2018).

182. P.-H. Chen, X. Zhu, H. Zhang, J. S. Turek, J. Chen, T. L. Willke, U. Hasson, P. J. Ramadge, A convolutional autoencoder for multi-subject fMRI data aggregation. arXiv:1608.04846 [stat.ML] (2016).

183. X. Du, C. Ma, G. Zhang, J. Li, Y.-K. Lai, G. Zhao, X. Deng, Y.-J. Liu, H. Wang, An efficient LSTM network for emotion recognition from multichannel EEG signals. *IEEE Trans. Affect. Comput.* **13**, 1528–1540 (2020).

184. I. Sosin, D. Kudenko, A. Shpilman, Continuous gesture recognition from sEMG sensor data with recurrent neural networks and adversarial domain adaptation, in *2018 15th International Conference on Control, Automation, Robotics and Vision (ICARCV)* (IEEE, 2018). pp. 1436–1441.

185. J. Liu, X. Gong, Attention mechanism enhanced LSTM with residual architecture and its application for protein-protein interaction residue pairs prediction. *BMC Bioinformatics* **20**, 609 (2019).

186. M. Ahmed, J. Islam, M. R. Samee, R. E. Mercer, Identifying protein-protein interaction using tree LSTM and structured attention, in *2019 IEEE 13th International Conference on Semantic Computing (ICSC)* (2019). pp. 224–231.

187. Y. Ganin, V. Lempitsky, Unsupervised domain adaptation by backpropagation. in *Proceedings of the 32nd International Conference on Machine Learning*, F. Bach, D. Blei, Eds. (PMLR, 07–09 Jul 2015) vol. 37 of *Proceedings of Machine Learning Research*, pp. 1180–1189.

188. M. Baktashmotlagh, M. Salzmann, U. Dogan, M. Kloft, F. Orabona, T. Tommasi, Distribution-matching embedding for visual domain adaptation. *J. Mach. Learn. Res.* **17**, 1–30 (2016).

189. J. Hoffman, E. Tzeng, T. Park, J.-Y. Zhu, P. Isola, K. Saenko, A. Efros, T. Darrell, CyCADA: Cycle-consistent adversarial domain adaptation, in *Proceedings of the 35th International Conference on Machine Learning*, J. Dy, A. Krause, Eds. (PMLR, 10–15 Jul 2018) vol. 80 of *Proceedings of Machine Learning Research*, pp. 1989–1998.

190. S. Rakshit, B. Banerjee, G. Roig, S. Chaudhuri, Unsupervised multi-source domain adaptation driven by deep adversarial ensemble learning, in *Pattern Recognition* (Springer International Publishing, 2019), pp. 485–498.

191. A. Gretton, K. Borgwardt, M. Rasch, B. Schölkopf, A. Smola, A kernel method for the two-sample-problem. *Adv. Neural Inf. Process. Syst.* **19**, (2007).

192. G. Kang, L. Jiang, Y. Yang, A. G. Hauptmann, Contrastive adaptation network for unsupervised domain adaptation, in *2019 IEEE/CVF Conference on Computer Vision and Pattern Recognition (CVPR)* (IEEE, 2019), pp. 4893–4902.

193. H. Guo, R. Pasunuru, M. Bansal, Multi-source domain adaptation for text classification via DistanceNet-bandits. *AAAI* **34**, 7830–7838 (2020).

194. B. B. Damodaran, B. Kellenberger, R. Flamary, D. Tuia, N. Courty, DeepJDOT: Deep joint distribution optimal transport for unsupervised domain adaptation. arXiv:1803.10081 [cs. CV] (2018).

195. M. Ghifary, W. B. Kleijn, M. Zhang, D. Balduzzi, W. Li, Deep reconstruction-classification networks for unsupervised domain adaptation, in *Computer Vision–ECCV 2016* (Springer International Publishing, 2016), pp. 597–613.

196. M. Ghifary, W. B. Kleijn, M. Zhang, D. Balduzzi, Domain generalization for object recognition with Multi-task autoencoders. arXiv:1508.07680 [cs.CV] (2015).

197. Y.-H. Tsai, W.-C. Hung, S. Schulter, K. Sohn, M.-H. Yang, M. Chandraker, Learning to adapt structured output space for semantic segmentation. arXiv:1802.10349 [cs.CV] (2018).

198. J.-Y. Zhu, T. Park, P. Isola, A. A. Efros, Unpaired image-to-image translation using cycle-consistent adversarial networks, in *2017 IEEE International Conference on Computer Vision (ICCV)* (IEEE, 2017), pp. 2223–2232.

199. S. Zhao, C. Lin, P. Xu, S. Zhao, Y. Guo, R. Krishna, G. Ding, K. Keutzer, CycleEmotionGAN: Emotional semantic consistency preserved CycleGAN for adapting image emotions. *AAAI* **33**, 2620–2627 (2019).
